# An in-silico planning study of stereotactic body radiation therapy for polymetastatic patients with more than ten extra-cranial lesions

**DOI:** 10.1016/j.phro.2024.100567

**Published:** 2024-03-03

**Authors:** Federico Iori, Nathan Torelli, Jan Unkelbach, Stephanie Tanadini-Lang, Sebastian M. Christ, Matthias Guckenberger

**Affiliations:** a Radiation Oncology Unit, Azienda USL-IRCCS di Reggio Emilia, 42122 Reggio Emilia, Italy; b Department of Radiation Oncology, University Hospital Zurich, University of Zurich, Rämistrasse 100, 8091 Zurich, Switzerland; c Clinical and Experimental Medicine PhD Program, Department of Biomedical, Metabolic, and Neural Sciences, University of Modena and Reggio Emilia, Modena, Italy

**Keywords:** Metastatic disease, Polymetastatic disease, Stereotactic Body Radiation Therapy, Radiation Therapy, Cancer, Treatment Planning, Study

## Abstract

**Background and purpose:**

Limited data is available about the feasibility of stereotactic body radiation therapy (SBRT) for treating more than five extra-cranial metastases, and almost no data for treating more than ten. The aim of this study was to investigate the feasibility of SBRT in this polymetatstatic setting.

**Materials and methods:**

Consecutive metastatic melanoma patients with more than ten extra-cranial metastases and a maximum lesion diameter below 11 cm were selected from a single-center prospective registry for this in-silico planning study. For each patient, SBRT plans were generated to treat all metastases with a prescribed dose of 5x7Gy, and dose-limiting organs (OARs) were analyzed. A cell-kill based inverse planning approach was used to automatically determine the maximum deliverable dose to each lesion individually, while respecting all OARs constraints.

**Results:**

A total of 23 polymetastatic patients with a medium of 17 metastases (range, 11–51) per patient were selected. SBRT plans with sufficient target coverage and respected OARs dose constraints were achieved in 16 out of 23 patients. In the remaining seven patients, the lungs V5Gy < 80 % and the liver D700 cm^3^ < 15Gy were most frequently the dose-limiting constraints. The cell-kill based planning approach allowed optimizing the dose administration depending on metastases total volume and location.

**Conclusion:**

This retrospective planning study shows the feasibility of definitive SBRT for 70% of polymetastatic patients with more than ten extra-cranial lesions and proposes the cell-killing planning approach as an approach to individualize treatment planning in polymetastatic patients’.

## Highlights

• Stereotactic body radiation therapy delivering 5x7Gy was feasible in 16 of 23 (70 %) patients with > 10 metastases.
• In six of the seven cases where normal tissue constraints called for lowered prescription doses, the lung and the liver were most often the dose-limiting organs.
• Cell-kill based planning was found to be a promising approach for high metastatic burden patients.

## Graphical abstract

**Figure:**
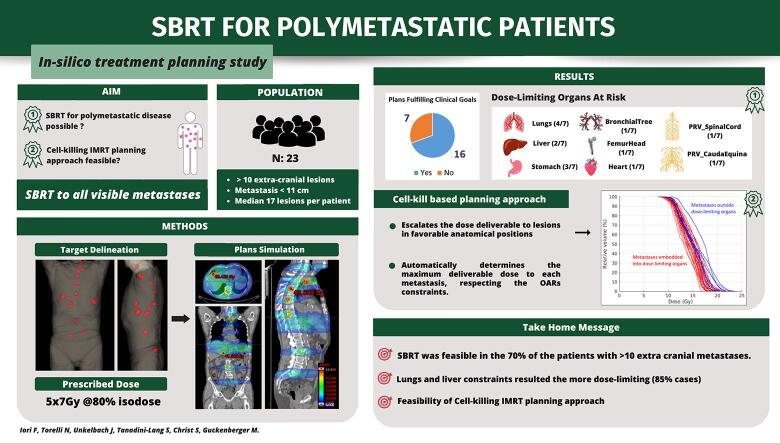

## 1 Introduction

Metastatic disease progression is responsible for most cancer-related deaths. The Spectrum Theory proposes that the neoplastic disease is a continuum, ranging between a localized and a polymetastatic state [1]. Thus, the oligometastatic condition is an intermediate cancer stage, with a limited metastatic capacity, where definitive local treatment of all macro-metastases combined with systematic therapy can improve patients’ prognosis [2]. The SABR-COMET trial was a proof-of-principle of this theory, which showed that local ablation of all metastases with stereotactic body radiation therapy (SBRT) can increase the 5-year overall survival (42 % vs 18 %) in oligometastatic patients with one to five lesions [3].

Differently from the oligometastatic condition, polymetastatic disease represents the final stage of cancer and it is usually managed with systemic therapies, only. Although the exact number of lesions to differentiate the polymetastatic from the oligometastatic setting is still debatable, there is a unanimous agreement that the polymetastatic condition is more aggressive with poor long-term survival [4], [5]. Hence, expanding and translating the concept of comprehensive metastases ablation into the polymetastatic setting, the combination of a metastases-targeting strategy with a systemic therapy, could be a valuable approach for polymetastatic patients. Since the metastasis-to-metastasis diffusion, i.e., the mushrooming of daughter metastases from a maternal one, is a key driver of cancer polymetastatic progression [6], treating all visible lesions with SBRT could potentially delay tumor dissemination, prolong the time to systemic therapies resistance development, and improve patients’ outcomes.

Despite most oligometastatic trials allowed the inclusion of patients with a maximum burden of three to five lesions, the actual number of treated metastases was only one or two in almost all studies [7]. Moreover, excluding brain metastases, no data is yet available to demonstrate the technical feasibility of comprehensive SBRT in the polymetastatic setting [8], [9]. The SABR-COMET-10 trial (*NCT03721341*) is evaluating the clinical benefit of ablating from four to ten metastasis, however, the study population does not encompass polymetastatic patients with more than ten lesions [10]. Thus, to the best of our knowledge, the ARREST trial (*NCT04530513*) is the only prospective study that goes beyond the oligometastatic setting, investigating the safety of SBRT in patients with a minimum of ten metastases [11].

In this in-silico treatment planning study, we therefore investigated the feasibility of SBRT for polymetastatic patients with more than ten extra-cranial lesions. Additionally, we proposed and investigated an alternative planning approach that departs from the concept that all metastases are treated with one homogeneous dose level but optimizes and maximizes the dose to each lesion individually, by minimizing the number of surviving cancer cells while respecting organs at risks (OARs) constraints.

## 2 Materials and methods

### 2.1 Study population

We searched the prospective metastatic melanoma patients’ database of the University Hospital Zürich to indentify patients for this study. The RATING guidelines were followed (RATING-score: 97 %; Supplementary_Material_A) [12]. This study was approved and authorized by the internal review board. Eligible patients had to present more than ten extra-cranial metastases at baseline and a maximum lesion diameter below 11 cm. We focused on melanoma patients because the latest ESMO guidelines recommend their enrollment in trials, especially when polymetastatic, due to the natural disease evolution and the limits of available systemic therapies [13].

Out of 488 patients, 25 presented a tumor burden with more than extra-cranial metastases. Of these, two patients were excluded because of lesions with a maximum diameter > 11 cm. This resulted in 23 consecutive patients for this planning study, with a median age of 70 years (range, 33–83 years) and a male and female prevalence of 74 % and 26 %, respectively. The total lesions number was 477, with a median number of 17 metastatic lesions per patient (range, 11–51). Metastases were in the thorax (207; 44 %), abdomen (168; 35 %), pelvis (44; 9 %), lower limbs (31; 7 %), head and neck (20; 4 %), and upper limbs (7; 1 %). The median individual metastasis volume was 4 cm^3^ (range, 2– 14 cm^3^), with a median GTV_all and PTV_all volumes of 82 cm^3^ (range 34–246 cm^3^) and 291 cm^3^ (range, 134–757 cm^3^), respectively. A median of five VMAT arcs, ranging from two to eight, and two isocenters per SBRT plan were used (Supplementary_Material_B for details).

### 2.2 Target delineation

Diagnostic computed tomography (CT) scans of every eligible patient were collected and imported into the ARIA oncology information system for medical and radiation oncology (Varian Medical Systems, Palo Alto, CA, USA). A radiation oncologist contoured each individual metastasis as a single Gross Tumor Volume (GTV; GTV_1, GTV_2, etc). All GTV’s were then merged using Boolean-Operator to create GTV_all. The Planning Target Volume (PTV) was created with an isotropic 5 mm margin expansion of GTV_all (PTV_all), as a minimum 5 mm margin is considered reasonable using single-isocenter VMAT for multiple targets [14]. PTV_all was cropped isotropically to 3 mm from the body surface if it expanded outside of the body contour.

### 2.3 Oars delineation

For OARs delineation, the DICOM files for every CT scan were exported into MIM Contour ProtégéAI (MIM Software Inc. Cleveland, OH, USA) where the following structures were automatically segmented: trachea, esophagus, bronchial tree, lungs, heart, brachial plexus, liver, stomach, duodenum, great vessels, kidneys, bowel, bladder, spinal cord, cauda equina and femur heads. Thereafter, all contours were manually reviewed, to adjust eventual imprecisions. A Planning Risk Volume (PRV) was defined for spinal cord and cauda equine, adopting an isotropic 0.2 cm margin expansion from the corresponding OAR, as performed in the SABR-COMET-10 trial [10].

### 2.4 Constraints

A literature search was performed to identify OARs tolerance doses in SBRT for metastatic disease. We evaluated and compared the five-fractions SBRT constraints of the SABR-COMET, the SABR-COMET-10, the ARREST trial, the NRG-BR001 trial, the NRG-BR002 trial, the UK 2022, and the 2021 guidelines of the France Society of Radiotherapy and Oncology (Supplementary_Material_C) [3], [10], [15], [16], [17], [18]. Consequently, we decided to adopt the tolerance doses reported in the Supplementary_Material_D for the targets and the OARs. All tolerance doses were used as hard constraints in our planning study. As irradiating large body volumes may impair the hematopoietic system, we aimed to estimate the hematopoietic bone marrow (HBM) dose. With the majority of HBM in the adults being located in vertebral bodies, pelvis bones, and femoral heads, we assumed these structures as a reasonable patients’ hematopoietic bone marrow approximation.

### 2.5 SBRT planning and dose prescription

SBRT plans were generated for each patient using the Eclipse Treatment Planning System (TPS) (Varian Medical Systems, Palo Alto, CA, USA), with a Volumetric Modulated Arc Therapy (VMAT) technique for a Varian TrueBeam treatment unit equipped with Millenium 120 MLC. The SBRT plans were firstly created using a single-isocenter approach and two VMAT arcs. However, considering the maximum fields’ extension (x_max: 14.6 cm; y_max: 35 cm), additional VMAT arcs were added and the final number of isocenters and arcs was thereby decided on a case-by-case basis, according to lesions spread in axial and sagittal direction. Dose calculation was performed with Acuros External Beam (Version 16.1.0) and plan optimization used the PO16.1.0 algorithm. The photon energy was set to 6 MV for all arcs.

SBRT was investigated with a schedule of 5x7Gy, as this is the minimum dose applied in the SABR-COMET-10 trial [10]. This dose was prescribed at the 80 % isodose level, such that the GTV_all was supposed to receive at least 113 % of the prescribed dose with a Maximum Point Dose (Dmax) up to 126 %. Dose-volume and mean dose objectives were used to achieve clinical goals for the OARs, whereas the normal tissue objective (NTO) was used along with ring structures around the PTV_all to achieve dose conformity. Simulated plans were reviewed by a medical physicist and a radiation oncologist for acceptance. SBRT plans were accepted if all clinical goals in the Supplementary_Material_D were met. Nonetheless, we also considered a Dmax up to 129 % acceptable in the GTV, and a conformity index up to 1.40 (defined as volume receiving the prescribed dose divided by the PTV_all volume), if all other clinical goals were respected, allowing also a limited compromise of PTV coverage.

If it was not possible to respect all OARs constraints for a prescribed dose to the PTV of 5x7Gy, we progressively decreased the dose per fraction in steps of 1 Gy for all PTVs, by progressively decreased the number of Monitor Units (MU), until all OARs constraints were met. Thereafter, these plans with reduced MU was re-optimized again to individualise the dose-limiting OARs dose constraints.

### 2.6 Cell-kill based planning approach

For patients where we did not achieve 5x7Gy due to violation of OAR constraints, an alternative planning approach was proposed, which aimed at minimizing the number of surviving tumor cells while keeping all OAR constraints. A linear-quadratic cell survival model was assumed [19], according to which the total number of surviving tumor cells is:

(1) $$\sum_{m=1}^{M}\sum_{i\in PTV_{m}}exp\left(\left(-\alpha d_{i}-\beta d_{i}^{2}\right)N_{f}\right)$$

where $d_{i}$ is the dose in voxel i, α and β are the radiosensitivity parameters, and we sum over all voxels $i\in PTV_{m}$ that belong to any of the metastases m∈{1,⋯,M} (with M being the total number of metastases). Minimizing the number of surviving clonogens in Equation (1) is mathematically equivalent to maximizing Niemierko’s tumor EUD [20]

(2) $$\mathrm{EUD}=\frac{1}{2}\left[-\frac{\alpha }{\beta }+\sqrt{{\left(\frac{\alpha }{\beta }\right)}^{2}-\frac{4}{\beta N_{f}}\text{ln}\left(\frac{1}{N_{\mathrm{PTV}}}\sum_{m=1}^{M}\sum_{i\in PTV_{m}}exp\left(\left(-\alpha d_{i}-\beta d_{i}^{2}\right)N_{f}\right)\right)}\right]$$

where $N_{\mathrm{PTV}}$ is the total number of voxels in the union of all metastases. Equation (2) was used as an objective function for IMRT planning, together with hard constraints to ensure that all OAR constraints are satisfied. To guarantee that an acceptable dose conformity is achieved, a dynamic conformity objective was implemented, based on a quadratic penalty function that penalize voxel doses above a given fraction of the PTV dose depending on the voxels distance from the PTV. As no prescribed dose is defined a priori for the PTV, Niemierko’s EUD was used as reference dose for the conformity objective.

The cell-kill based planning approach, that is implemented into our in-house research optimization software, assuming$N_{f}$ = 5 fractions and radiobiological parameters of $\alpha =0.29Gy^{-1}$ and $\beta =0.029Gy^{-2}$, which corresponds to α/β=10 and a surviving fraction of 50 % at 2 Gy. Additional details regarding the optimization method are reported in Supplementary_Material_E.

## 3 Results

### 3.1 Treatment plans

Out of the 23 patients, treatment plans of 16 patients (70 %) respected all clinical goals of OARs sparing and simultaneously achieved acceptable target coverage of all metastases with 5x7Gy, being feasible (hereafter referred to as *accepted plans: AP*). Treatment plans of seven patients were rejected (RP) as they required OARs constraints violation to satisfy targets goals with 5x7Gy, being unfeasible. The median number of metastases per patient was 26 (range, 11–51) for the RP and 15 (range, 11–28) for the AP, respectively (p-value < 0.05; while the median GTV_all volume was 156 cm^3^ (range, 55–246 cm^3^) and 55 cm^3^ (range, 35–235 cm^3^) for RP and AP, respectively (p-value < 0.05). No statistically significant (p-value < 0.05) difference was observed in terms of anatomical lesions dispositions or involved anatomical sites between AP and RP (Table 1). For AP, the HBM V2Gy was 4 cm^3^ (range, 3–11 cm^3^), representing a median of 44 % of HBM volume (range, 20–77 %), respectively. The dose was verified for three patients in the Delta4 phantom and evaluated using a global gamma agreement index with 3 % dose difference and 2 mm distance to agreements. The dosimetric and geometric accuracy of the delivery was excellent with all three plans passing with 100 % gamma agreement index. The detailed evaluation of 1 patient is in Supplementary_Material_G. The average beam on time was 12 min (range 8–16 min).

**Table 1:** Comparison of the AP and RP, considering the patients’ characteristics, the metastases number and their disposition across the body as well as the infiltrated structures. *Statistically significant (p-value < 0.05) difference (for further information about the p-values calculation and the statistical tests adopted, please see the Supplementary_Material_F). ^#^We refer to the percentage of the organ that receive the prescribe dose, i.e., that is included in the PTV.** Percentage of total volume Abbreviation: AP: Accepted Plans; RP: Rejected Plans. The median MU number refers to the median MU per fraction, per plan.

	AP	RP
**Patients Characteristics**		
Patients Number	16	7
Age	64 years (38 – 83 years)	70 years (33–78 years)
Sex	12 males – 5 females	5 males – 2 females
Median Lesions Number*	15 (range 11–28)	26 (range 11–51)

**Lesion Number and anatomical disposition**		
Total Number	263 (100 %)	214 (100 %)
H&N	8 (3 %)	12 (6 %)
Thorax	107 (41 %)	100 (46 %)
Abdomen	84 (32 %)	84 (39 %)
Pelvis	32 (12 %)	12 (6 %)
Extremity	32 (12 %)	6 (3 %)

**Involved Anatomical Sites**		
Adrenal gland	12 (4 %)	1 (1 %)
Bone	29 (11 %)	64 (30 %)
Heart	0 (0 %)	1 (1 %)
Intercostal	1 (1 %)	0 (0 %)
Intraperitoneal	12 (4 %)	1 (1 %)
Lymph nodes	68 (26 %)	27 (12 %)
Liver	32 (13 %)	59 (27 %)
Lung	51 (19 %)	53 (24 %)
Muscle	8 (3 %)	0 (0 %)
Retroperitoneal	7 (3 %)	0 (0 %)
Spleen	4 (2 %)	7 (3 %)
Subcutaneous	22 (8 %)	1 (1 %)
Paravertebral	17 (6 %)	0 (0 %)

**Hematopoietic Bone Marrow**		
HBC Total Volume	1508 cm^3^ (998–1954 cm^3^)	1667 cm^3^ (1209–1932 cm^3^)
V2Gy**	44 % (20–78 %)	23 % (13–87 %)
Dmean	4 Gy (3–11 Gy)	4 Gy (2–16 Gy)

**Plan Characteristics**		
Median GTV_all*	55 cm^3^ (35––235 cm^3^)	156 cm^3^ (55–246 cm^3^)
Median Treated Volume*	17 cm^3^ (118– 435 cm^3^)	497 cm^3^ (236– 757 cm^3^)
Median MU	6938 (3390 – 9570)	9357 (4494 – 13808)
Median Arcs Number	5 (2–8)	6 (3–8)
Median Isocenters Number	2 (1–3)	2 (1–3)
Median Infiltrated Lungs’ Volume	0 % (0–1 %)	0.1 % (0–3 %)
Median Treated Lungs’ Volume^#^	0.4 % (0– 1 %)	2.3 % (0–7 %)
Median Infiltrated Liver’s Volume	0.3 % (0–5 %)	0 % (0– 5 %)
Median Treated Liver’s Volume^#^	1.3 % (0–10 %)	0.3 % (0– 21 %)

### 3.2 Dose-limiting organs and MU reduction

With refence to the seven RP, the volumetric constraints for the parallel OARs lung and liver were dose-limiting for six out of seven patients. In particular, the lung V5Gy, the stomach D0.1 cm^3^, the liver D700 cm^3^ were dose-limiting in four, three and two out of seven of the RP, respectively, while the lungs D1500 cm^3^ and the point dose of PRV_SpinalCord, PRV_CaudaEquina, heart, bronchial tree, and femur heads were dose-limiting only in one case each.

After the iterative reduction of monitor units in the RP, the maximum deliverable dose (MDD) fulfilling all target and OARs clinical goals were found (Table 2).

**Table 2:** First column: RP identifier; Second and Third column: dose-limiting organs with the corresponding dose-limiting constraints; Fourth column: highest target dose deliverable respecting all clinical goal that was reached by the iterative reduction of Monitor Units (i.e., dose per fraction). For further information, please see the Supplementary_Material_E.

Patient	Organs	Dose-Limiting Constraint for higher doses	Maximum Deliverable Dose
Patient_6	Lungs-GTV_all	V5.0 Gy	5x2Gy
Patient_8	Lungs-GTV_all	V5.0 Gy	5x2Gy
Patient_9	Lungs-GTV_all	D1500 cm^3^	5x4Gy
Patient_12	Lungs-GTV_all	V5.0 Gy	5x4Gy
		V13.5 Gy	
Patient_14	Liver-GTV_all	D700 cm^3^	5x6Gy
	Stomach	D0.1 cm^3^	
	Femur Head Right	D10 cm^3^	
Patient_17	Liver-GTV_all	D700 cm^3^	5x3Gy
Patient_19	Lungs-GTV_all	V5.0 Gy	5x5Gy
	PRV_SpinalCord	D0.1 cm^3^	
	PRV_CaudaEquina	D0.1 cm^3^	

### 3.3 Treatment plans obtained using the cell-kill based planning approach

**Fig. 1:**
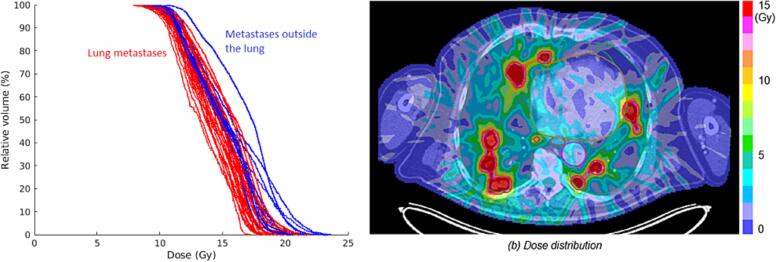
(a) Dose-volume histograms of the cell-kill based planning approach, for the PTV of each metastasis in Patient_6. Lesions within the lung are depicted using red lines, whereas lesions outside the lung which do not contribute to the lung dose are shown with blue lines. (b) Dose distribution obtained using the cell-kill based planning approach for Patient_6. The contours for the PTV (red), lungs (green), heart (orange), great vessel (violet) and esophagus (brown) are also illustrated. This refers to a single fraction out of 5 and it shows how it is possible to deliver with this apporach higher doses to metastases outside the lungs. The figure shows how it is possible to treat concurrently diverse metastases with different dose without violating clinical goals, depending on their anatomical location. (For interpretation of the references to colour in this figure legend, the reader is referred to the web version of this article.)

Fig. 1 shows the dose-volume histograms and the dose distribution for the plan obtained using the cell-kill based planning approach for Patient_6. Although an exact quantitative comparison between this plan and the treatment plan generated in Eclipse was not possible due to differences in both the dose calculation and optimization algorithms, the cell-kill based approach was able to automatically determine the maximum dose that could be delivered to each metastasis individually, while preserving OARs function. Particularly, similar minimum doses of approximately 8–9 Gy were delivered to all metastases in Patient_6, where the value of the minimum dose was limited by the dose constraints on the healthy lung. However, inhomogeneous doses were delivered within each lesion, with the mean and maximum doses varying in between the individual metastases up to 3.4 Gy and 6.5 Gy, respectively. The cell-kill based planning approach exploited that lesions located in favorable anatomical locations could be irradiated to a higher radiation dose than others. In particular, for Patient_6, lesions located far from the lung and that did not contribute to the lung dose (depicted as blue lines in Fig. 1a), tended to receive a larger dose compared to lung metastases (depicted as red lines in Fig. 1a). Similar results were obtained for Patient_17 (Supplementary_Material_E*)*.

## 4 Discussion

This retrospective in-silico planning study of polymetastatic patients investigated the feasibility of definitive SBRT targeting more than ten extra-cranial metastases. We were able to demonstrate that SBRT of all visible metastases with a prescribed radiation dose of 35 Gy in five fractions was feasible for the majority of the patients, with definitive SBRT for 11 to 28 metastases per patient. To our knowledge, this is one of the first studies addressing the dosimetric feasibility of delivering SBRT in patients with more than ten metastases and the first performed on a truly polymetastatic patient population. Only for 7 out of 23 patients, it was not possible to deliver this target dose to all metastases without violating OAR constraints. These patients were characterized by a higher metastatic tumor burden and the prevalence of a higher number and volume of metastases in the thorax and in the abdomen. The lungs and liver OAR constraints were dose-limiting in the vast majority of these patients, especially due to the violation of lung V5Gy and of liver D700 cm^3^. Our results can form the basis for future clinical evaluation of SBRT in patients with more than ten metastases.

Only two very early clinical trials are currently evaluating feasibility and safety of SBRT in polymetastatic patients. SABR-COMET-10 is a randomized phase III trial, which compares SBRT to all visible metastases in patients with four to ten metastases in addition to standard-of-care (SoC) systemic therapy, with SoC systemic therapy alone (*NCT03721341*). Patients are treated with three fractionation schedules of 1x20Gy, 3x10Gy or 5x7Gy, the latter selected to minimize the risks of toxicity [10]. Primary endpoint is overall survival. However, the SABR-COMET-10 trial does not enroll patients with more than ten metastases. Hence, the ARREST study is the only prospective phase I trial (*NCT04530513*) testing definitive SBRT in patients with tumor burden beyond oligometastatic condition [11]. In the ARREST study, patients are enrolled with a minimum of 11 distant metastases to evaluate SBRT safety. The prescribed dose is not decided a priori but determined with incremental weekly fractions of 6 Gy up to a maximum dose of 30 Gy, delivered to a maximum of 50 targets. The preliminary planning study showed that such schedule was feasible for patients with metastases smaller than 3 cm in diameter. However, this study was based only on five CTs (five patients) without initial metastases, as all targets were added to these CTs by randomly contouring them with reasonable sizes and anatomical positions [21].

Considering the feasibility endpoint of this in-silico planning study, it is important to account that the OAR constraints mostly derived from single-target SBRT or conventionally fractionated RT, with the dose conversion performed using the linear-quadratic model [22]. The SABR-COMET-3 and the NRG-BR001 study demonstrated the constraints validity for the oligometastatic setting; however, it remains unknown whether these tolerance doses are valid for multiple-target SBRT. Irradiating large volumes could entail a lymph-myeloid suppression, with the potential consequence of preventing patients further systemic therapy and ultimately worsening their prognosis [23], [24]. In our planning study, we did not perform bone marrow sparing but recorded dose expose to estimate hematological toxicity [25].

For multi-site SBRT for polymetastatic cancer patients, it is a priori unclear what radiotherapy dose can or should be prescribed to each metastasis. Generating RT plans with a fixed prescribed dose and iterative de-escalating until all clinical goals are met, allows determining of the maximum dose which can be safety delivered to all metastases. However, such a process is time-consuming; moreover, metastases located in more favorable anatomical locations could potentially be treated to a higher dose compared to lesions embedded within or close to dose-limiting OARs. To address this problem, we proposed and demonstrated an alternative planning approach, which aimed to minimize the number of surviving cancer cells while preserving organs’ function. Such a cell-kill based approach was previously suggested by Niemierko for solid tumors [20] but did not find widespread application in treatment planning. In this study, we revisit the concept and show its potential value for polymetastatic cancer patients. It represents an addition to the conventional planning objectives such as quadratic penalty function that may be useful for polymetastaic patients to automatically determine the maximum radiation dose that can be delivered to each individual metastasis, saving planning time and concurrently satisfying all OAR constraints.

This study presents some limitations. First, a fixed GTV-to-PTV margin expansion was used independently from the risk of moving tumors, which is most likely not realistic in a clinical setting and might impair clinical application. This is particularly relevant when treating multiple targets with a single isocenter, as it may increase the risk of geographical missing. Application of metastases-individual safety margins could address this issue, which was outside the scope of this planning study. Second, it recruited a limited population composed by melanoma patients, only. Third, the high number of MU per plan entail a prolonged treatment time that may result challenging for a polymetastatic patient. Finally, although this is not expected to significantly affect the dosimetric results, this work was performed based on diagnostic rather than planning CT scans.

In conclusion, this in-silico planning study indicates that definitive SBRT of polymetastatic patients with minimum 11 metastases might be feasible in a majority of unselected metastatic melanoma patients, broadening the perspective of the curative RT beyond the oligometastatic disease. Additionally, novel approaches in SBRT planning using e.g., cell-kill based objective functions might further help improving the safety and the feasibility of a SBRT treatment for patients with higher lesion burden or problematic metastases location that prevents the administration of local ablative treatments with conventional planning approaches.

## CRediT authorship contribution statement

**Federico Iori:** Conceptualization, Project administration, Data curation, Software, Formal analysis, Investigation, Methodology, Writing – original draft, Writing – review & editing. **Nathan Torelli:** Conceptualization, Project administration, Data curation, Software, Methodology, Writing – original draft, Writing – review & editing. **Jan Unkelbach:** Conceptualization, Project administration, Supervision, Software, Writing – review & editing. **Stephanie Tanadini-Lang:** Supervision, Data curation, Software, Methodology, Writing – review & editing. **Sebastian M. Christ:** Software, Formal analysis, Writing – review & editing. **Matthias Guckenberger:** Conceptualization, Project administration, Supervision, Investigation, Methodology, Writing – review & editing.

## Declaration of competing interest

The authors declare the following financial interests/personal relationships which may be considered as potential competing interests: Federico Iori, Nathan Torelli, Jan Unkelbach and Sebastian Christ declare no conflicts of interest. Stephanie Tanadini-Lang declares that her husband works at Varian/Siemens Healthineers. Matthias Guckenberger declares research support from ViewRay, Varian, AstraZeneca, and of being on AstraZeneca Advisory Board.
