# Supplementary material for: An in-silico planning study of stereotactic body radiation therapy for polymetastatic patients with more than ten extra-cranial lesions
Source: Phys Imaging Radiat Oncol. 2024 Mar 3;30:100567. doi: 10.1016/j.phro.2024.100567 (PMC10950805; doi:10.1016/j.phro.2024.100567)
Supplement: Supplementary data 2 [file mmc2.pdf]

Supplementary Material B

Summary

a. Metastases disposition

b. Clinical Goals

c. Dose Volume Endpoints

d. Overview of all patients plans

e. Accepted Plans and PTV Compromises

f. Constrains Violation in Rejected Plans

g. Monitor Units Reduction

h. Hematopoietic Bone Marrow

a. Metastases Disposition

The selected patients presented several metastasis across the body. In the figure below (Table 1), we summarize lesions' location, describing the anatomical regions that were infiltrated. To visualize in detail the target for each patient, please refer to the Paragraph c and d of this Appendix_B.

Lesions Location	N (%)
**Adrenal Gland**	13 (2.73%)
**Bone**	93 (19.49%)
**Heat**	1 (0.21%)
**Intercostal**	1 (0.21%)
**Intraperitoneal**	13 (2.73%)
**Liver**	91 (19.08%)
**Lymph Node**	95 (19.92%)
**Lungs**	104 (21.80%)
**Muscle**	8 (1.68%)
**Paravertebral**	17 (3.56%)
**Retroperitoneal**	7 (1.47%)
**Spleen**	11(2.30%)
**Subcutaneous**	23 (4.82%)

Table 1:*The table shows the position of the lesions of all patients across the body*

Below, the summary of patients' metastatic burden. First column: patient identifier; second column: total number of irradiated metastases across the body for each patient; third, fourth, and fifth column: number of irradiated metastases in lungs, liver, and spine. *Planning criteria for radiotherapy (RT) delivery of 35Gy in 5 fractions (Fx) NOT fulfilled.

Patient identifier	Total number of metastases	Number of lung metastases	Number of liver metastases	Number of spine metastases
Patient 1	17	0	0	0
Patient 2	13	0	0	0
Patient 3	11	8	0	0
Patient_4	14	2	1	0

Patient_5	13	10	0	1
Patient_6*	37	23	0	6
Patient_7	23	0	9	5
Patient_8*	26	18	0	0
Patient_9*	11	9	0	0
Patient_10	15	0	0	0
Patient_11	15	0	0	0
Patient_12*	18	3	2	1
Patient_13	18	2	3	8
Patient_14*	24	0	14	2
Patient_15	15	1	5	0
Patient_16	12	0	3	0
Patient_17*	51	0	43	0
Patient_18	15	6	3	1
Patient_19*	47	0	0	31
Patient_20	28	10	6	1
Patient_21	22	1	0	0
Patient_22	15	2	1	1
Patient_23	17	9	1	2

b. Clinical Goals Adopted

**TARGET**	
**Structure**	**Clinical Goal**
**GTV_all**	V 110.0% > 95%
	D 0.1 cm^3 < 126%
	D 0.1 cm^3 > 123%
**PTV_all**	CI ≥ 1.00
	CI < 1.20
	V 100.0% > 95%
**OARs**	
**Structure**	**Clinical Goal in 5 fraction SBRT**
**Bladder**	D 15.0 cm^3 < 27.50 Gy
	D 0.1 cm^3 < 38.00 Gy
**Bowel**	D 0.1 cm^3 < 38.00 Gy
	D 20 cm^3 < 29.00 Gy
**BrachialPlexus_L**	D 3.0 cm^3 < 27.00 Gy
	D 0.1 cm^3 < 32.00Gy
**BrachialPlexus_R**	D 3.0 cm^3 < 27.00 Gy
	D 0.1 cm^3 < 32.00 Gy
**BronchialTree**	D 5.0 cm^3 < 32.00 Gy
	D 0.1 cm^3 < 38.00 Gy
**Duodenum**	D 10 cm^3 < 21.00 Gy
	D 0.1 cm^3 < 35.00 Gy
**Esophagus**	D 0.1 cm^3 < 35.00 Gy
**FemurHead_L**	D 10 cm^3 < 30.00 Gy
**FemurHead_R**	D 10 cm^3 < 30.00 Gy
**GreatVessel**	D 0.1 cm^3 < 53.00 Gy
**Heart**	D 0.1 cm^3 < 38.00 Gy
	D 15.0 cm^3 < 32.00 Gy

**Kidneys – GTV_all**	D 200.0 cm^3 < 17.50 Gy
**Liver – GTV_all**	Dmean < 18.00 Gy
	D 700 cm^3 < 15 Gy
**Lungs – GTV_all**	V 5.00 Gy < 80 %
	V 20 Gy < 15.0 %
	V 13.50 Gy < 37.00 %
	D 1500.0 cm^3 < 12.50 Gy
**PRV_CaudaEquina**	D 0.1 cm^3 < 31.50 Gy
**PRV_SpinaCord**	D 0.1 cm^3 < 28.00 Gy
**Rectum**	D 0.1 cm^3 < 38.00 Gy
**Stomach**	D 10 cm^3 < 25.00 Gy
	D 0.1 cm^3 < 32.00 Gy
**Trachea**	D cm^3 < 38.00 Gy

c. Dose Volume Endpoint

The median CI amounted to 1.16 (range 1.00-1.40). The median Dmax value was 44.42 Gy (range 43.54-45.75 Gy). A median number of 5 arcs (range 2-8) per plan was used, with a median number of 2 isocenters and a median of 7156.9 MU (range 3390.4 -3807.9 MU) per plan. The table below (Table 2) shows the targets and organs at risk' dose-volume endpoints. From left to right, it is illustrated the structures' name, the clinical goal, the dose median value, the range of variability, the first quartile (Q1), and the third quartile (Q3). NOTE:* A patient had a metastasis inside the left femur head and this is the reason of the upper bund value.

Table 2

**Target**					
**Structure**	**Clinical Goal**	**Median**	**Range**	**Q1**	**Q3**
**GTV_all**	V 110.0% > 95%	97.84%	91.79-100 %	96.76%	99.24 %
	123% < D 0.1 cm^3 < 126%	125.26 %	91.80-125.89 %	123 %	124.69 %
**PTV_all**	1.00 ≤ CI < 1.20	1.16	1-1.38	1.08	1.19
	V 100.0% > 95%	95.42 %	95.01-99.50 %	95.01 %	95.81 %
**OARs**					
**Structure**	**Clinical Goal**	**Median**	**Range**	**Q1**	**Q3**
**Bladder**	D 15.0 cm^3	2.17 Gy	0.00-18.70 Gy	0.08 Gy	6.94 Gy
	D 0.1 cm^3	3.31 Gy	0.00-28.73 Gy	0.08 Gy	11.82 Gy
**Bowel**	D 0.1 cm^3	35.51 Gy	13.29-37.98 Gy	13.29 Gy	37.19 Gy
	D 20 cm^3	17.65 Gy	3.03-28.24 Gy	13.52 Gy	22.87 Gy
**BrachialPlexus_L**	D 3.0 cm^3	1.07 Gy	0.08-24.54 Gy	0.44 Gy	4.22 Gy
	D 0.1 cm^3	3.93 Gy	0.21-30.28 Gy	1.40 Gy	7.04 Gy
**BrachialPlexus_R**	D 3.0 cm^3	1.16 Gy	0.02-25.14 Gy	0.39 Gy	2.59 Gy
	D 0.1 cm^3	1.88 Gy	0.17-30.55 Gy	0.97 Gy	14.65 Gy
**BronchialTree**	D 5.0 cm^3	11.47 Gy	0.27-32.72 Gy	8.57 Gy	32.72 Gy
	D 0.1 cm^3	21.59 Gy	0.4-36.29 Gy	14.24 Gy	35.05 Gy
**Duodenum**	D 10 cm^3	6.67 Gy	0.00-19.60 Gy	2.79 Gy	16.29 Gy
	D 0.1 cm^3	20.21Gy	0.00-33.82 Gy	8.09 Gy	25.03 Gy
**Esophagus**	D 0.1 cm^3	29.15 Gy	4.64-34.55 Gy	21.83 Gy	33.63 Gy
**FemurHead_L**	D 10 cm^3	1.87 Gy	0.00-40.53 Gy*	0.07 Gy	7.92 Gy
**FemurHead_R**	D 10 cm^3	1.56 Gy	0.00-30 Gy	0.04 Gy	10.48 Gy
**GreatVessels**	D 0.1 cm^3	39.15 Gy	9.36-43.31 Gy	35.26 Gy	41.57 Gy
**Heart**	D 0.1 cm^3	25.66 Gy	0.31-40.76 Gy	17.01 Gy	35.90 Gy

	D 15.0 cm^3	19.81Gy	0.92-34.15 Gy	9.95 Gy	22.78 Gy
**Kidneys – GTV_all**	D 200.0 cm^3	2.11 Gy	0.10-9.68 Gy	0.10 Gy	4.66 Gy
**Liver – GTV_all**	Dmean	9.71 Gy	0.24-28.26 Gy	7.02 Gy	13.13 Gy
	D 700 cm^3	9.52 Gy	0.21-32.28 Gy	3.91 Gy	12.31 Gy
**Lungs – GTV_all**	V 5.00 Gy	63.74 %	1.14-99.82 %	1.14 %	78.85 %
	V 20.00 Gy	6.59 %	0.03-54.36 %	2.28 %	12.11 %
	V13.50 Gy	17.45 %	0.23-80.35 %	10.32 %	80.35 %
	D 1500.0 cm^3	7.79 Gy	0.33-24.47 Gy	4.85 Gy	9.93 %
**PRV_CaudaEquina**	D 0.1 cm^3	10.92 Gy	0.35-37.04 Gy	2.06 Gy	24.29 Gy
**PRV_SpinalCord**	D 0.1 cm^3	23.27 Gy	4.48-38.61 Gy	20.42 Gy	24.34 Gy
**Rectum**	D 0.1 cm^3	5.30 Gy	0.00-31.33 Gy	0.08 Gy	16.3 Gy
**Stomach**	D 0.1 cm^3	20.03 Gy	0.43-30.54 Gy	13.98 Gy	30.54 Gy
	D 10 cm^3	16.53 Gy	0.37-33.36 Gy	10.89 Gy	20.79 Gy
**Trachea**	D 0.1 cm^3	18.30 Gy	0.67-37.81 Gy	10.39 Gy	37.81 Gy

d. Overview of all patients plans

Patients	Metastatic burden	Location	OAR	Lesions	Clinical Goal	DV E	Status
Patient_1	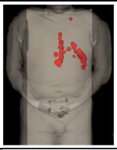	H: 0	Lungs	0	V 5.00 Gy	58.39 %	✓
		T: 7			V 20 Gy	6.75%	✓
		A: 10			V 13.50 Gy	17.45 %	✓
		P: 0			D 1500.0 cm^3	5.11 Gy	✓
		E: 0	Liver	0	D 700 cm^3	9.95 Gy	✓
		Mets: 17			Dmean	8.89 Gy	✓
			Spine	0	PSC_D 0.1 cm^3	23.00 Gy	✓
					PCE_D 0.1 cm^3	0.76 Gy	✓
Patient_2	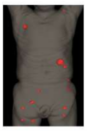	H: 0	Lungs	0	V 5.00 Gy	4.63 %	✓
		T: 1			V 20 Gy	0.04 %	✓
		A: 2			V 13.50 Gy	0.27 %	✓
		P: 3			D 1500.0 cm^3	0.44 Gy	✓
		E: 7	Liver	0	D 700 cm^3	0.25 Gy	✓
		Mets: 13			Dmean	0.42 Gy	✓
			Spine	0	PSC_D 0.1 cm^3	4.48 Gy	✓
					PCE_D 0.1 cm^3	8.12 Gy	✓
Patient_3	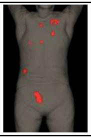	H: 0	Lungs	8	V 5.00 Gy	43.44 %	✓
		T: 9			V 20 Gy	5.05 %	✓
		A: 1			V 13.50 Gy	9.74 %	✓
		P: 1			D 1500.0 cm^3	5.29 Gy	✓
		E: 0	Liver	0	D 700 cm^3	0.36 Gy	✓
		Mets: 11			Dmean	0.96 Gy	✓
			Spine	0	PSC_D 0.1 cm^3	21.26 Gy	✓
					PCE_D 0.1 cm^3	6.97 Gy	✓
Patient_4	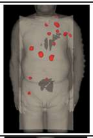	H: 1	Lungs	2	V 5.00 Gy	62.84 %	✓
		T: 6			V 20 Gy	6.59 %	✓
		A: 4			V 13.50 Gy	17.31 %	✓
		P: 1			D 1500.0 cm^3	4.93 Gy	✓
		E: 2	Liver	1	D 700 cm^3	7.70 Gy	✓
		Mets: 14			Dmean	9.71 Gy	✓
			Spine	0	PSC_D 0.1 cm^3	17.97 Gy	✓
					PCE_D 0.1 cm^3	21.92 Gy	✓
Patient_5	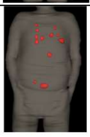	H: 0	Lungs	10	V 5.00 Gy	77.34 %	✓
		T: 10			V 20 Gy	12.30 %	✓
		A: 1			V 13.50 Gy	25.71 %	✓
		P: 2			D 1500.0 cm^3	7.79 Gy	✓
		E: 0	Liver	0	D 700 cm^3	3.24 Gy	✓
		Mets: 13			Dmean	3.97 Gy	✓
			Spine	1	PSC_D 0.1 cm^3	19.23 Gy	✓
					PCE_D 0.1 cm^3	26.54 Gy	✓
Patient_6	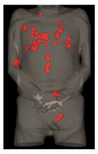	H: 1	Lungs	23	V 5.00 Gy	97.51 %	✗
		T: 28			V 20 Gy	54.36 %	✗
		A: 4			V 13.50 Gy	80.35 %	✗
		P: 3			D 1500.0 cm^3	24.47 Gy	✗
		E: 1	Liver	0	D 700 cm^3	9.52 Gy	✓
		Mets: 37			Dmean	9.06 Gy	✓
			Spine	6	PSC_D 0.1 cm^3	22.94 Gy	✓
					PCE_D 0.1 cm^3	22.70 Gy	✓
Patient_7	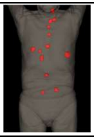	H: 1	Lungs	0	V 5.00 Gy	33.30 %	✓
		T: 5			V 20 Gy	2.22 %	✓
		A: 13			V 13.50 Gy	6.50 %	✓
		P: 4			D 1500.0 cm^3	4.58 Gy	✓
		E: 0	Liver	9	D 700 cm^3	11.20 Gy	✓
		Mets: 23			Dmean	10.73 Gy	✓
			Spine	5	PSC_D 0.1 cm^3	25.26 Gy	✓
					PCE_D 0.1 cm^3	31.27 Gy	✓
Patient_8		H: 0	Lungs	18	V 5.00 Gy	95.15 %	✗
		T: 25			V 20 Gy	44.48 %	✗

	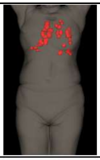	A: 1			V 13.50 Gy	78.89 %	✗
		P: 0			D 1500.0 cm^3	18.33 Gy	✗
		E: 0	Liver	0	D 700 cm^3	1.29 Gy	✓
					Dmean	5.15 Gy	✓
		Mets: 26	Spine	0	PSC_D 0.1 cm^3	23.94 Gy	✓
					PCE_D 0.1 cm^3	0.61 Gy	✓
Patient_9	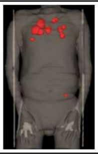	H: 0	Lungs	9	V 5.00 Gy	56.14 %	✓
		T: 10			V 20 Gy	30.45 %	✗
		A: 0			V 13.50 Gy	43.49 %	✗
		P: 1			D 1500.0 cm^3	18.88 Gy	✗
		E: 0	Liver	0	D 700 cm^3	0.24 Gy	✓
					Dmean	0.27 Gy	✓
		Mets: 11	Spine	0	PSC_D 0.1 cm^3	22.62 Gy	✓
					PCE_D 0.1 cm^3	0.08 Gy	✓
Patient_10	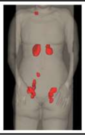	H: 1	Lungs	0	V 5.00 Gy	1.14 %	✓
		T: 0			V 20 Gy	0.08 %	✓
		A: 2			V 13.50 Gy	0.23 %	✓
		P: 3			D 1500.0 cm^3	0.33 %	✓
		E: 9	Liver	0	D 700 cm^3	12.18 Gy	✓
					Dmean	13.29 Gy	✓
		Mets: 15	Spine	0	PSC_D 0.1 cm^3	24.84 Gy	✓
					PCE_D 0.1 cm^3	10.92 Gy	✓
Patient_11	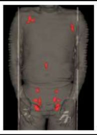	H: 0	Lungs	0	V 5.00 Gy	20.15 %	✓
		T: 5			V 20 Gy	0.19 %	✓
		A: 1			V 13.50 Gy	1.31 %	✓
		P: 2			D 1500.0 cm^3	2.32 Gy	✓
		E: 7	Liver	0	D 700 cm^3	0.35 Gy	✓
					Dmean	0.37 Gy	✓
		Mets: 15	Spine	0	PSC_D 0.1 cm^3	6.67 Gy	✓
					PCE_D 0.1 cm^3	12.29 Gy	✓
Patient_12	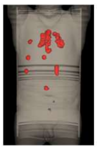	H: 0	Lungs	3	V 5.00 Gy	92.96 %	✗
		T: 14			V 20 Gy	36.47 %	✗
		A: 4			V 13.50 Gy	59.69 %	✗
		P: 0			D 1500.0 cm^3	15.63 Gy	✗
		E: 0	Liver	2	D 700 cm^3	11 Gy	✓
					Dmean	9.18 Gy	✓
		Mets: 18	Spine	1	PSC_D 0.1 cm^3	23.75 Gy	✓
					PCE_D 0.1 cm^3	28.25 Gy	✓
Patient_13	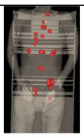	H: 1	Lungs	2	V 5.00 Gy	79.20 %	✓
		T: 8			V 20 Gy	8.08 %	✓
		A: 4			V 13.50 Gy	19.88 %	✓
		P: 5			D 1500.0 cm^3	8.55 Gy	✓
		E: 0	Liver	3	D 700 cm^3	10.86 Gy	✓
					Dmean	13.35 Gy	✓
		Mets: 18	Spine	8	PSC_D 0.1 cm^3	24.73 Gy	✓
					PCE_D 0.1 cm^3	16.23 Gy	✓
Patient_14	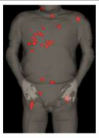	H: 2	Lungs	0	V 5.00 Gy	78.49 %	✓
		T: 3			V 20 Gy	3.01 %	✓
		A: 13			V 13.50 Gy	10.90 %	✓
		P: 3			D 1500.0 cm^3	5.60 %	✓
		E: 3	Liver	14	D 700 cm^3	17.23 Gy	✗
					Dmean	17.62 Gy	✓
		Mets: 24	Spine	2	PSC_D 0.1 cm^3	27.04 Gy	✓
					PCE_D 0.1 cm^3	16.89 Gy	✓
Patient_15	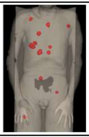	H: 0	Lungs	1	V 5.00 Gy	62.80%	✓
		T: 6			V 20 Gy	3.55 %	✓
		A: 6			V 13.50 Gy	11.46 %	✓
		P: 1			D 1500.0 cm^3	6.77 Gy	✓
		E: 2	Liver	5	D 700 cm^3	12.45 Gy	✓
					Dmean	13.23 Gy	✓
		Mets: 15	Spine	0	PSC_D 0.1 cm^3	20.03 Gy	✓
					PCE_D 0.1 cm^3	2.84 Gy	✓
Patient_16	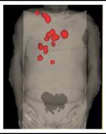	H: 1	Lungs	0	V 5.00 Gy	77.94 %	✓
		T: 5			V 20 Gy	8.58 %	✓
		A: 5			V 13.50 Gy	29.38 %	✓
		P: 1			D 1500.0 cm^3	9.38 Gy	✓
		E: 0	Liver	3	D 700 cm^3	13.67 Gy	✓
					Dmean	13.03 Gy	✓
		Mets: 12	Spine	0	PSC_D 0.1 cm^3	23.27 Gy	✓
					PCE_D 0.1 cm^3	0.54 Gy	✓
Patient_17	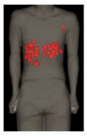	H: 0	Lungs	0	V 5.00 Gy	20.15 %	✓
		T: 1			V 20 Gy	5.79 %	✓
		A: 50			V 13.50 Gy	11.05 %	✓
		P: 0			D 1500.0 cm^3	1.50 Gy	✓
		E: 0	Liver	43	D 700 cm^3	32.28 Gy	✗
					Dmean	28.26 Gy	✗
		Mets: 51	Spine	0	PSC_D 0.1 cm^3	21.88 Gy	✓
					PCE_D 0.1 cm^3	1.17 Gy	✓
Patient_18	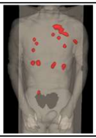	H: 0	Lungs	6	V 5.00 Gy	78.74 %	✓
		T: 8			V 20 Gy	5.03 %	✓
		A: 6			V 13.50 Gy	14.53 %	✓
		P: 1			D 1500.0 cm^3	14.53 %	✓
		E: 0	Liver	3	D 700 cm^3	8.54 Gy	✓
					Dmean	9.18 Gy	✓
		Mets: 15	Spine	1	PSC_D 0.1 cm^3	15.52 Gy	✓
					PCE_D 0.1 cm^3	1.28 Gy	✓
Patient_19	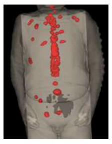	H: 9	Lungs	0	V 5.00 Gy	99.82 %	✗
		T: 19			V 20 Gy	6.38 %	✓
		A: 12			V 13.50 Gy	19.01 %	✓
		P: 5			D 1500.0 cm^3	9.36 Gy	✓
		E: 2	Liver	0	D 700 cm^3	9.03 Gy	✓
					Dmean	10.20 Gy	✓
		Mets: 47	Spine	31	PSC_D 0.1 cm^3	38.61 Gy	✗
					PCE_D 0.1 cm^3	37.04 Gy	✗
Patient_20	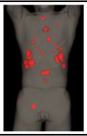	H: 1	Lungs	10	V 5.00 Gy	79.24 %	✓
		T: 13			V 20 Gy	11.92 %	✓
		A: 11			V 13.50 Gy	28.12 %	✓
		P: 0			D 1500.0 cm^3	10.51 Gy	✓
		E: 3	Liver	6	D 700 cm^3	14.86 Gy	✓
					Dmean	15.57 Gy	✓
		Mets: 28	Spine	1	PSC_D 0.1 cm^3	6.45 Gy	✓
					PCE_D 0.1 cm^3	3.68 Gy	✓
Patient_21		H: 0	Lungs	1	V 5.00 Gy	43.28 %	✓
		T: 6			V 20 Gy	1.54 %	✓
		A: 9			V 13.50 Gy	4.38 %	✓

	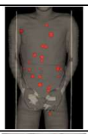	P: 6			D 1500.0 cm^3	6.65 Gy	✓
		E: 1	Liver	0	D 700 cm^3	8.52 Gy	✓
		Mets: 22			Dmean	8.89 Gy	✓
			Spine	0	PSC_D 0.1 cm^3	23.45 Gy	✓
					PCE_D 0.1 cm^3	11.31 Gy	✓
Patient_22	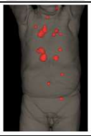	H: 1	Lungs	2	V 5.00 Gy	63.74 %	✓
		T: 7			V 20 Gy	8.97 %	✓
		A: 5			V 13.50 Gy	27.24 %	✓
		P: 2			D 1500.0 cm^3	10.46 Gy	✓
		E: 0	Liver	1	D 700 cm^3	13.30 Gy	✓
		Mets: 15			Dmean	13.94 Gy	✓
			Spine	0	PSC_D 0.1 cm^3	23.58 Gy	✓
					PCE_D 0.1 cm^3	6.21 Gy	✓
Patient_23	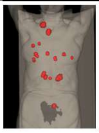	H: 1	Lungs	9	V 5.00 Gy	69.93 %	✓
		T: 11			V 20 Gy	12.58 %	✓
		A: 4			V 13.50 Gy	29.27 %	✓
		P: 1			D 1500.0 cm^3	7.92 Gy	✓
		E: 0	Liver	1	D 700 cm^3	4.57 Gy	✓
		Mets: 17			Dmean	11.26 Gy	✓
			Spine	2	PSC_D 0.1 cm^3	23.75 Gy	✓
					PCE_D 0.1 cm^3	25.88 Gy	✓

Characteristics of all patients' plans. First column: patient identifier; second column: location of target lesions across the body; third and fourth column: number of metastases in lung, liver and spine. For these tree OARs, the clinical goals, their dose volume endpoints, and values in the finally accepted treatment plans are reported, including the status whether planning objectives were (met: ✓- violated: ✗); DVE: dose-volume endpoint; Met: metastases; PCE: PRV_CaudaEquina; PSC: PRV_SpinalCord. Note: the lung and the liver endpoint are referred to Lungs-GTV_all and Liver-GTV_all, respectively

e. Accepted Plans Dose Volume Endpoint and PTV Compromises

Out of 23 plans, 16 plans were accepted as they met the clinical goals and it was possible to administer prescribed dose (i.e., 35 Gy in 5 fractions at 80% of the isodose line).

In some cases it was necessary to compromise a minimal part of the PTV_all adjacent to critical OARs, still respecting the target goals; however, all lesions had to receive the prescribe dose and no lesion was exempted.

In the next pages, we report all the CT_targets used to simulate the plans for both the accepted plans and the rejected ones. For each plan, we illustrate the PTV_all disposition across the body, as well as the dose volume endpoints and the Monitor Units count. Additionally, we show for how many lesions a compromise was performed – i.e., under-dosed lesions-, explaining which were the dose limiting organs that imposed the coverage compromise. We also report the number of isocenters that we used, as well as the total number of fields required for each plan- amid brackets are reported the number of fields for each isocenter in detail-.

Patient_1

N_isocenters = 1	Lesions = 17	Monitor Units: 3390.3
N_fields = 2	Under-dosed Lesions = 0	

The Figure 1 below illustrate the spatial localization across the body of the PTV_all. In this case, no lesion was under-dosed to achieve an adequate plan. In Table 3 the dose-volume endpoints are reported.

Figure 1

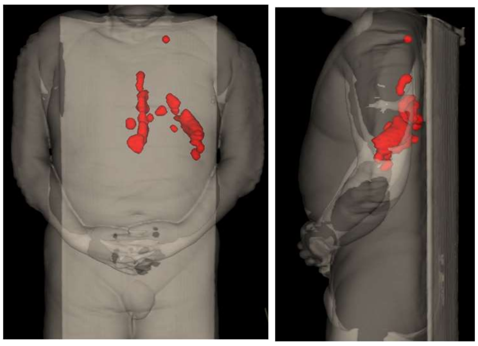

Table 3

Dose Volume Endpoints		
**Structure**	**Clinical Goal**	**Value**
**GTV_all**	V 110.0% > 95%	99.50%
	123% < D 0.1 cm^3 < 126%	123.02%
**PTV_all**	1.00 ≤ CI < 1.20	1.09
	V 100.0% > 95%	95.70%
**Bladder**	D 15.0 cm^3	0.10 Gy
	D 0.1 cm^3	0.12 Gy
**Bowel**	D 0.1 cm^3	33.91 Gy
	D 20 cm^3	16.84 Gy
**BrachialPlexus_L**	D 3.0 cm^3	0.67 Gy
	D 0.1 cm^3	1.38 Gy
**BrachialPlexus_R**	D 3.0 cm^3	0.43 Gy
	D 0.1 cm^3	0.72 Gy
**BronchialTree**	D 5.0 cm^3	8.44 Gy
	D 0.1 cm^3	14.64 Gy
**Duodenum**	D 10 cm^3	15.51 Gy
	D 0.1 cm^3	18.42 Gy
**Esophagus**	D 0.1 cm^3	20.92 Gy
**FemurHead_L**	D 10 cm^3	0.06 Gy
**FemurHead_R**	D 10 cm^3	0.05 Gy

**GreatVessels**	D 0.1 cm^3	42.02 Gy
**Heart**	D 0.1 cm^3	17.62 Gy
	D 15.0 cm^3	14.56 Gy
**Kidneys – GTV_all**	D 200.0 cm^3	4.09 Gy
**Liver – GTV_all**	Dmean	8.89 Gy
	D 700 cm^3	9.95 Gy
**Lungs – GTV_all**	V 5.00 Gy	58.39 %
	V 20.00 Gy	6.75%
	V13.50 Gy	17.45 %
	D 1500.0 cm^3	5.11 Gy
**PRV_CaudaEquina**	D 0.1 cm^3	0.76 Gy
**PRV_SpinalCord**	D 0.1 cm^3	23.00 Gy
**Rectum**	D 0.1 cm^3	0.08 Gy
**Stomach**	D 0.1 cm^3	20.03 Gy
	D 10 cm^3	16.58 Gy
**Trachea**	D 0.1 cm^3	8.59 Gy

Patient_2

**N_isocenters = 3**	**Lesions = 13**	**Monitor Units: 9486.4**
**N_fields = 7 (2+2+3)**	**Under-dosed Lesions = 0**	

The Figure 2 below illustrate the spatial localization across the body of the PTV_all. In this case, no lesion was under-dosed to achieve an adequate plan. In Table 4 the dose-volume endpoints are reported.

Figure 2

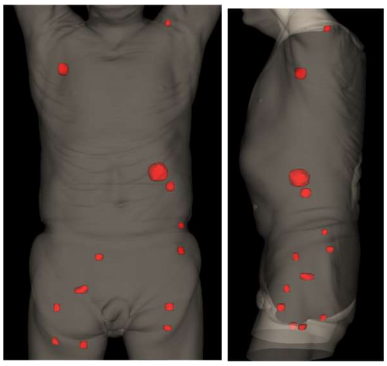

Table 4

**Dose Volume Endpoints**		
**Structure**	**Clinical Goal**	**Value**
**GTV_all**	V 110.0% > 95%	99.66%
	123% < D 0.1 cm^3 < 126%	123.02 %
**PTV_all**	1.00 ≤ CI < 1.20	1.04
	V 100.0% > 95%	95.45 %
**Bladder**	D 15.0 cm^3	1.90 Gy
	D 0.1 cm^3	4.30 Gy
**Bowel**	D 0.1 cm^3	37.98 Gy
	D 20 cm^3	27.93 Gy
**BrachialPlexus_L**	D 3.0 cm^3	1.28 Gy
	D 0.1 cm^3	3.93 Gy
**BrachialPlexus_R**	D 3.0 cm^3	1.16 Gy
	D 0.1 cm^3	1.67 Gy
**BronchialTree**	D 5.0 cm^3	1.61 Gy
	D 0.1 cm^3	3.38 Gy
**Duodenum**	D 10 cm^3	4.60 Gy
	D 0.1 cm^3	8.99 Gy
**Esophagus**	D 0.1 cm^3	4.64 Gy

**FemurHead_L**	D 10 cm^3	10.40 Gy
**FemurHead_R**	D 10 cm^3	16.60 Gy
**GreatVessels**	D 0.1 cm^3	9.36 Gy
**Heart**	D 0.1 cm^3	0.31 Gy
	D 15.0 cm^3	0.28 Gy
**Kidneys – GTV_all**	D 200.0 cm^3	4.35 Gy
**Liver – GTV_all**	Dmean	0.42 Gy
	D 700 cm^3	0.25 Gy
**Lungs – GTV_all**	V 5.00 Gy	4.63 %
	V 20.00 Gy	0.04 %
	V13.50 Gy	0.27 %
	D 1500.0 cm^3	0.44 Gy
**PRV_CaudaEquina**	D 0.1 cm^3	8.12 Gy
**PRV_SpinalCord**	D 0.1 cm^3	4.48 Gy
**Rectum**	D 0.1 cm^3	27.89 Gy
**Stomach**	D 0.1 cm^3	6.18 Gy
	D 10 cm^3	1.54 Gy
**Trachea**	D 0.1 cm^3	4.60 Gy

Patient_3

**N_isocenters = 2**	**Lesions = 11**	**Monitor Units: 8699.4**
**N_fields = 5 (2+3)**	**Under-dosed Lesions = 0**	

The Figure 3 below illustrate the spatial localization across the body of the PTV_all. In this case, no lesion was under-dosed to achieve an adequate plan. In Table 5 the dose-volume endpoints are reported.

Figure 3

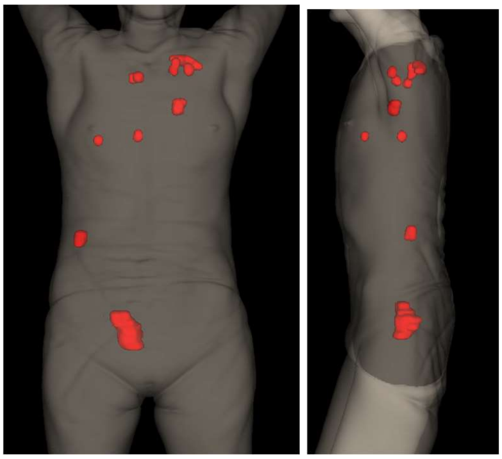

Table 5

**Dose Volume Endpoints**		
**Structure**	**Clinical Goal**	**Value**
**GTV_all**	V 110.0% > 95%	100%
	123% < D 0.1 cm^3 < 126%	123.01%
**PTV_all**	1.00 ≤ CI < 1.20	1.06 %
	V 100.0% > 95%	97.90 %
**Bladder**	D 15.0 cm^3	7.04 Gy
	D 0.1 cm^3	20.49 Gy
**Bowel**	D 0.1 cm^3	37.15 Gy
	D 20 cm^3	24.39 Gy
**BrachialPlexus_L**	D 3.0 cm^3	0.72 Gy
	D 0.1 cm^3	6.08 Gy
**BrachialPlexus_R**	D 3.0 cm^3	0.42 Gy
	D 0.1 cm^3	1.88 Gy
**BronchialTree**	D 5.0 cm^3	11.47 Gy
	D 0.1 cm^3	35.83 Gy

**Duodenum**	D 10 cm^3	3.17 Gy
	D 0.1 cm^3	7.18 Gy
**Esophagus**	D 0.1 cm^3	23.00 Gy
**FemurHead_L**	D 10 cm^3	5.57 Gy
**FemurHead_R**	D 10 cm^3	12.39 Gy
**GreatVessels**	D 0.1 cm^3	42.07 Gy
**Heart**	D 0.1 cm^3	14.76 Gy
	D 15.0 cm^3	8.04 Gy
**Kidneys – GTV_all**	D 200.0 cm^3	1.55 Gy
**Liver – GTV_all**	Dmean	0.96 Gy
	D 700 cm^3	0.36 Gy
**Lungs – GTV_all**	V 5.00 Gy	43.44 %
	V 20.00 Gy	5.05 %
	V13.50 Gy	9.74 %
	D 1500.0 cm^3	5.29 Gy
**PRV_CaudaEquina**	D 0.1 cm^3	6.97 Gy
**PRV_SpinalCord**	D 0.1 cm^3	21.26 Gy
**Rectum**	D 0.1 cm^3	31.33 Gy
**Stomach**	D 0.1 cm^3	3.74 Gy
	D 10 cm^3	2.82 Gy
**Trachea**	D 0.1 cm^3	21.99 Gy

Patient_4

**N_isocenters = 2**	**Lesions = 14**	**Monitor Units: 7156.9**
**N_fields = 5 (2+3)**	**Under-dosed Lesions = 0**	

The Figure 4 below illustrate the spatial localization across the body of the PTV_all. In this case, no lesion was under-dosed to achieve an adequate plan. In Table 6 the dose-volume endpoints are reported.

Figure 4

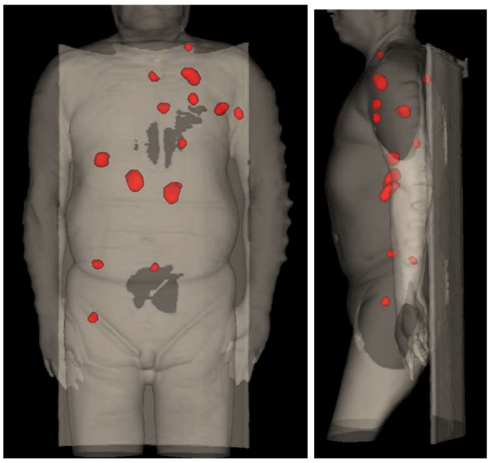

Table 6

**Dose Volume Endpoints**		
**Structure**	**Clinical Goal**	**Value**
**GTV_all**	V 110.0% > 95%	95.87 %
	123% < D 0.1 cm^3 < 126%	123.56%
**PTV_all**	1.00 ≤ CI < 1.20	1.16
	V 100.0% > 95%	95.01%
**Bladder**	D 15.0 cm^3	3.25 Gy
	D 0.1 cm^3	3.80 Gy
**Bowel**	D 0.1 cm^3	29.06 Gy
	D 20 cm^3	19.31 Gy
**BrachialPlexus_L**	D 3.0 cm^3	3.22 Gy
	D 0.1 cm^3	4.38 Gy
**BrachialPlexus_R**	D 3.0 cm^3	2.35 Gy
	D 0.1 cm^3	4.85 Gy
**BronchialTree**	D 5.0 cm^3	9.16 Gy

	D 0.1 cm^3	35.80 Gy
Duodenum	D 10 cm^3	16.82 Gy
	D 0.1 cm^3	29.92 Gy
Esophagus	D 0.1 cm^3	33.93 Gy
FemurHead_L	D 10 cm^3	1.48 Gy
FemurHead_R	D 10 cm^3	15.23 Gy
GreatVessels	D 0.1 cm^3	39.61 Gy
Heart	D 0.1 cm^3	23.23 Gy
	D 15.0 cm^3	15.74 Gy
Kidneys – GTV_all	D 200.0 cm^3	2.30 Gy
Liver – GTV_all	Dmean	9.71 Gy
	D 700 cm^3	7.70 Gy
Lungs – GTV_all	V 5.00 Gy	62.84 %
	V 20.00 Gy	6.59 %
	V13.50 Gy	17.31 %
	D 1500.0 cm^3	4.93 Gy
PRV_CaudaEquina	D 0.1 cm^3	21.92 Gy
PRV_SpinalCord	D 0.1 cm^3	17.97 Gy
Rectum	D 0.1 cm^3	3.09 Gy
Stomach	D 0.1 cm^3	15.63 Gy
	D 10 cm^3	11.18 Gy
Trachea	D 0.1 cm^3	15.67 Gy

Patient_5

**N_isocenters = 2**	**Lesions = 13**	**Monitor Units: 5919.2**
**N_fields = 5 (3+2)**	**Under-dosed Lesions = 0**	

The Figure 5 below illustrate the spatial localization across the body of the PTV_all. In this case, no lesion was under-dosed to achieve an adequate plan. In Table 7 the dose-volume endpoints are reported.

Figure 5

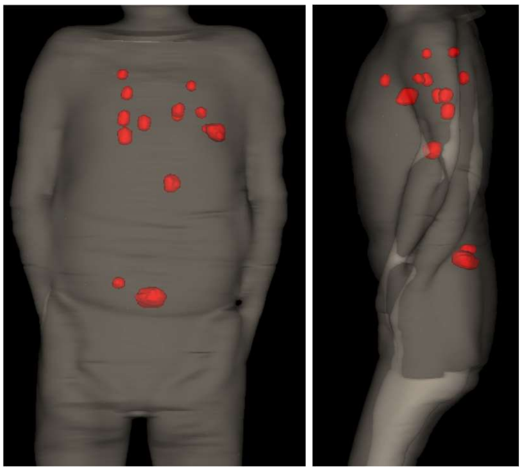

Table 7

Dose Volume Endpoints		
**Structure**	**Clinical Goal**	**Value**
GTV_all	V 110.0% > 95%	100%
	123% < D 0.1 cm^3 < 126%	124.05 %
PTV_all	1.00 ≤ CI < 1.20	1.19
	V 100.0% > 95%	99.50 %
Bladder	D 15.0 cm^3	9.75 Gy
	D 0.1 cm^3	12.14 Gy
Bowel	D 0.1 cm^3	23.27 Gy
	D 20 cm^3	13.60 Gy
BrachialPlexus_L	D 3.0 cm^3	0.33 Gy
	D 0.1 cm^3	0.51 Gy
BrachialPlexus_R	D 3.0 cm^3	0.45 Gy
	D 0.1 cm^3	0.58 Gy

**BronchialTree**	D 5.0 cm^3	17.60 Gy
	D 0.1 cm^3	34.57 Gy
**Duodenum**	D 10 cm^3	5.35 Gy
	D 0.1 cm^3	6.34 Gy
**Esophagus**	D 0.1 cm^3	29.28 Gy
**FemurHead_L**	D 10 cm^3	0.31 Gy
**FemurHead_R**	D 10 cm^3	0.36 Gy
**GreatVessels**	D 0.1 cm^3	30.29 Gy
**Heart**	D 0.1 cm^3	32.17 Gy
	D 15.0 cm^3	19.92 Gy
**Kidneys – GTV_all**	D 200.0 cm^3	0.60 Gy
**Liver – GTV_all**	Dmean	3.97 Gy
	D 700 cm^3	3.24 Gy
**Lungs – GTV_all**	V 5.00 Gy	77.34 %
	V 20.00 Gy	12.30 %
	V13.50 Gy	25.71 %
	D 1500.0 cm^3	7.79 Gy
**PRV_CaudaEquina**	D 0.1 cm^3	26.54 Gy
**PRV_SpinalCord**	D 0.1 cm^3	19.23 Gy
**Rectum**	D 0.1 cm^3	1.51 Gy
**Stomach**	D 0.1 cm^3	18.30 Gy
	D 10 cm^3	13.62 Gy
**Trachea**	D 0.1 cm^3	7.41 Gy

Patient_7

**N_isocenters = 3**	**Lesions = 23**	**Monitor Units: 6794.8**
**N_fields = 6 (2+2+2)**	**Under-dosed Lesions = 4 (2 PRV_SpinalCord; 1 Esophagus;1 PRV_CaudaEquina)**	

The Figure 6 below illustrate the spatial localization across the body of the PTV_all. In this case, no lesion was under-dosed to achieve an adequate plan. In Table 8 the dose-volume endpoints are reported.

Figure 6

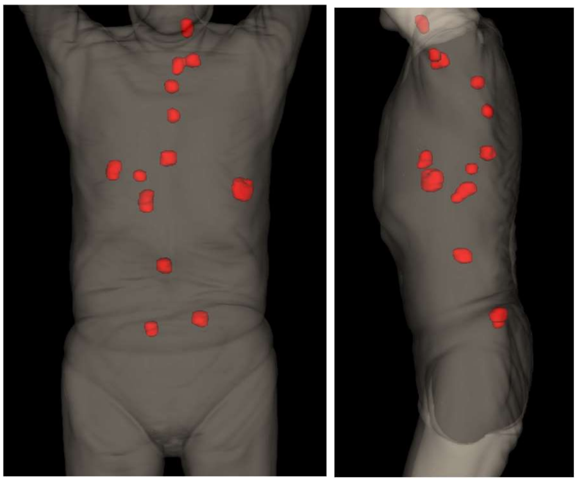

Table 8

**Dose Volume Endpoints**		
**Structure**	**Clinical Goal**	**Value**
**GTV_all**	V 110.0% > 95%	98.24 %
	123% < D 0.1 cm^3 < 126%	125.91 %
**PTV_all**	1.00 ≤ CI < 1.20	1.38
	V 100.0% > 95%	95.01 %
**Bladder**	D 15.0 cm^3	0.50 Gy
	D 0.1 cm^3	0.64 Gy
**Bowel**	D 0.1 cm^3	36.77 Gy
	D 20 cm^3	22.01 Gy
**BrachialPlexus_L**	D 3.0 cm^3	13.37 Gy
	D 0.1 cm^3	29.77 Gy

Note: * The patient had a history surgery and the duodenum was exported.

Below there are displayed the lesions for which a compromise was required; the 35 Gy isodose is highlighted):

Esophagus

**BrachialPlexus_R**	D 3.0 cm^3	2.68 Gy
	D 0.1 cm^3	6.61 Gy
**BronchialTree**	D 5.0 cm^3	9.23 Gy
	D 0.1 cm^3	13.84 Gy
**Duodenum**	D 10 cm^3	*
	D 0.1 cm^3	*
**Esophagus**	D 0.1 cm^3	34.14 Gy
**FemurHead_L**	D 10 cm^3	0.23 Gy
**FemurHead_R**	D 10 cm^3	.24 Gy
**GreatVessels**	D 0.1 cm^3	20.03 Gy
**Heart**	D 0.1 cm^3	17.84 Gy
	D 15.0 cm^3	14.33 Gy
**Kidneys – GTV_all**	D 200.0 cm^3	1.81 Gy
**Liver – GTV_all**	Dmean	10.73 Gy
	D 700 cm^3	11.20 Gy
**Lungs – GTV_all**	V 5.00 Gy	33.30 %
	V 20.00 Gy	2.22 %
	V13.50 Gy	6.50 %
	D 1500.0 cm^3	4.58 Gy
**PRV_CaudaEquina**	D 0.1 cm^3	31.27 Gy
**PRV_SpinalCord**	D 0.1 cm^3	25.26 Gy
**Rectum**	D 0.1 cm^3	1.91 Gy
**Stomach**	D 0.1 cm^3	23.18 Gy
	D 10 cm^3	19.53 Gy
**Trachea**	D 0.1 cm^3	35.34 Gy

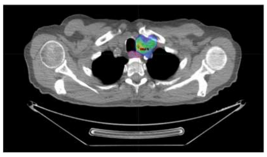

PRV_SpinaCord

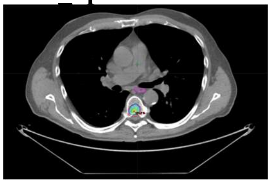

PRV_SpinaCord

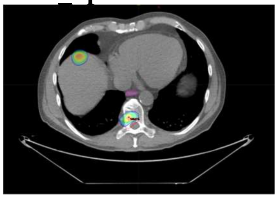

PRV_CaudaEquina

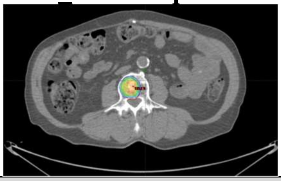

Patient_10

**N_isocenters = 3**	**Lesions = 15**	**Monitor Unit: 8451.0**
**N_fields = 6 (2+2+2)**	**Under-dosed Lesions = 1 (Stomach)**	

The Figure 7 below illustrate the spatial localization across the body of the PTV_all. In this case, no lesion was under-dosed to achieve an adequate plan. In Table 9 the dose-volume endpoints are reported.

Figure 7

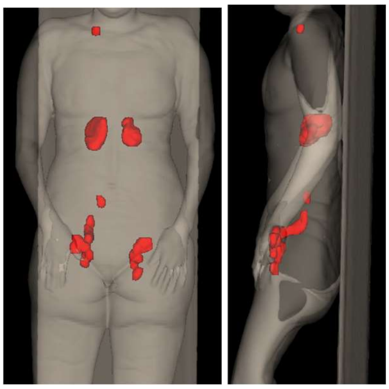

Table 9

**Dose Volume Endpoints**		
**Structure**	**Clinical Goal**	**Value**
**GTV_all**	V 110.0% > 95%	97.08 %
	123% < D 0.1 cm^3 < 126%	123.82 %
**PTV_all**	1.00 ≤ CI < 1.20	1.06
	V 100.0% > 95%	95.01 %
**Bladder**	D 15.0 cm^3	18.07 Gy
	D 0.1 cm^3	20.95 Gy
**Bowel**	D 0.1 cm^3	37.22 Gy

Below there are displayed the lesion were a compromise was required; the 35 Gy isodose is highlighted):

Stomach

	D 20 cm^3	26.02 Gy
**BrachialPlexus_L**	D 3.0 cm^3	0.17 Gy
	D 0.1 cm^3	2.99 Gy
**BrachialPlexus_R**	D 3.0 cm^3	0.16 Gy
	D 0.1 cm^3	0.96 Gy
**BronchialTree**	D 5.0 cm^3	0.27 Gy
	D 0.1 cm^3	0.40 Gy
**Duodenum**	D 10 cm^3	2.95 Gy
	D 0.1 cm^3	19.45 Gy
**Esophagus**	D 0.1 cm^3	18.92 Gy
**FemurHead_L**	D 10 cm^3	7.21 Gy
**FemurHead_R**	D 10 cm^3	19.33 Gy
**GreatVessels**	D 0.1 cm^3	36.65 Gy
**Heart**	D 0.1 cm^3	10.63 Gy
	D 15.0 cm^3	2.80 Gy
**Kidneys – GTV_all**	D 200.0 cm^3	1.23 Gy
**Liver – GTV_all**	Dmean	13.29 Gy
	D 700 cm^3	12.18 Gy
**Lungs – GTV_all**	V 5.00 Gy	1.14 %
	V 20.00 Gy	0.08 %
	V13.50 Gy	0.23 %
	D 1500.0 cm^3	0.33 Gy
**PRV_CaudaEquina**	D 0.1 cm^3	10.92 Gy
**PRV_SpinalCord**	D 0.1 cm^3	24.84 Gy
**Rectum**	D 0.1 cm^3	17.53 Gy
**Stomach**	D 0.1 cm^3	29.32 Gy
	D 10 cm^3	24.54 Gy
**Trachea**	D 0.1 cm^3	8.20 Gy

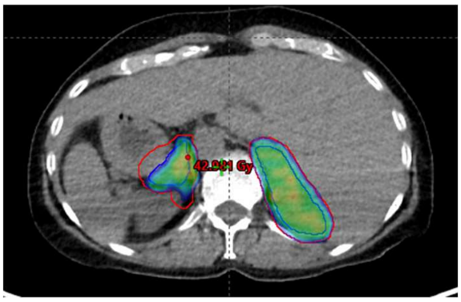

We compromise the lesion close to the stomach. It is important to remark that this simulation CT has a patient positioned feet-first.

Patient_11

**N_isocenters = 2**	**Lesions = 15**	**Monitor Units: 6627.8**
**N_fields = 4 (2+2)**	**Under-dosed Lesions = 0**	

The Figure 8 below illustrate the spatial localization across the body of the PTV_all. In this case, no lesion was under-dosed to achieve an adequate plan. In Table 10 the dose-volume endpoints are reported.

Figure 8

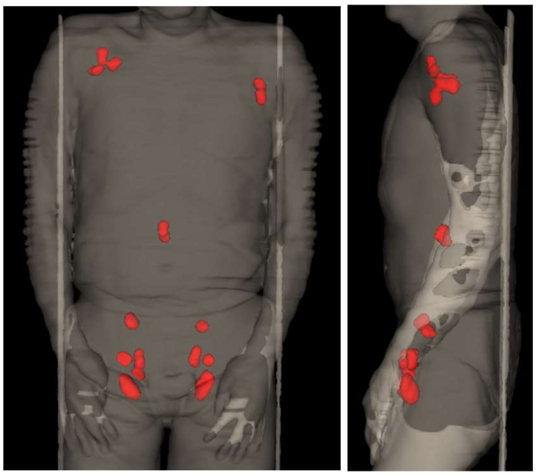

Table 10

**Dose Volume Endpoints**		
**Structure**	**Clinical Goal**	**Value**
**GTV_all**	V 110.0% > 95%	99.60 %
	123% < D 0.1 cm^3 < 126%	123.12 %
**PTV_all**	1.00 ≤ CI < 1.20	1.19
	V 100.0% > 95%	97.40 %
**Bladder**	D 15.0 cm^3	16.96 Gy

	D 0.1 cm^3	23.31 Gy
Bowel	D 0.1 cm^3	36.40 Gy
	D 20 cm^3	21.54 Gy
BrachialPlexus_L	D 3.0 cm^3	0.37 Gy
	D 0.1 cm^3	1.42 Gy
BrachialPlexus_R	D 3.0 cm^3	0.31 Gy
	D 0.1 cm^3	0.98 Gy
BronchialTree	D 5.0 cm^3	2.89 Gy
	D 0.1 cm^3	3.87 Gy
Duodenum	D 10 cm^3	16.09 Gy
	D 0.1 cm^3	30.50 Gy
Esophagus	D 0.1 cm^3	6.32 Gy
FemurHead_L	D 10 cm^3	7.92 Gy
FemurHead_R	D 10 cm^3	6.08 Gy
GreatVessels	D 0.1 cm^3	39.15 Gy
Heart	D 0.1 cm^3	1.40 Gy
	D 15.0 cm^3	0.92 Gy
Kidneys – GTV_all	D 200.0 cm^3	0.99 Gy
Liver – GTV_all	Dmean	0.37 Gy
	D 700 cm^3	0.35 Gy
Lungs – GTV_all	V 5.00 Gy	20.15 %
	V 20.00 Gy	0.19 %
	V13.50 Gy	1.31 %
	D 1500.0 cm^3	2.32 Gy
PRV_CaudaEquina	D 0.1 cm^3	12.29 Gy
PRV_SpinalCord	D 0.1 cm^3	6.76 Gy
Rectum	D 0.1 cm^3	19.40 Gy
Stomach	D 0.1 cm^3	0.43 Gy
	D 10 cm^3	0.49 Gy
Trachea	D 0.1 cm^3	6.21 Gy

Patient_13

**N_isocenters = 2**	**Lesions = 18**	**Monitor Units: 7606.1**
**N_fields = 7 (4+3)**	**Under-dosed Lesions = 5 (3 PRV_SpinalCord; 1 Esophagus; 1 Stomach)**	

The Figure 9 below illustrate the spatial localization across the body of the PTV_all. In this case, no lesion was under-dosed to achieve an adequate plan. In Table 11 the dose-volume endpoints are reported.

Figure 9

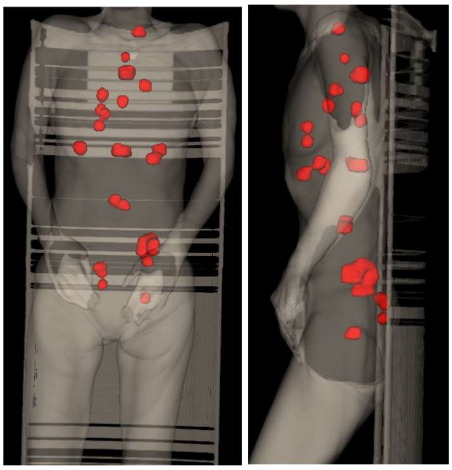

Table 11

Dose Volume Endpoints		
**Structure**	**Clinical Goal**	**Value**
GTV_all	V 110.0% > 95%	97.84 %
	123% < D 0.1 cm^3 < 126%	123.01 %
PTV_all	1.00 ≤ CI < 1.20	1.16
	V 100.0% > 95%	95.49 %
Bladder	D 15.0 cm^3	8.89 Gy
	D 0.1 cm^3	16.34 Gy

Below there are displayed the lesions for which a compromise was required; the 35 Gy isodose is highlighted):

PRV_SpinalCord

**Bowel**	D 0.1 cm^3	37.36 Gy
	D 20 cm^3	23.72 Gy
**BrachialPlexus_L**	D 3.0 cm^3	6.56 Gy
	D 0.1 cm^3	30.28 Gy
**BrachialPlexus_R**	D 3.0 cm^3	2.93 Gy
	D 0.1 cm^3	7.22 Gy
**BronchialTree**	D 5.0 cm^3	16.80 Gy
	D 0.1 cm^3	35.36 Gy
**Duodenum**	D 10 cm^3	11.50 Gy
	D 0.1 cm^3	22.17 Gy
**Esophagus**	D 0.1 cm^3	33.23 Gy
**FemurHead_L**	D 10 cm^3	13.63 Gy
**FemurHead_R**	D 10 cm^3	3.41 Gy
**GreatVessels**	D 0.1 cm^3	37.83 Gy
**Heart**	D 0.1 cm^3	35.26 Gy
	D 15.0 cm^3	22.86 Gy
**Kidneys – GTV_all**	D 200.0 cm^3	2.49 Gy
**Liver – GTV_all**	Dmean	13.35 Gy
	D 700 cm^3	10.86 Gy
**Lungs – GTV_all**	V 5.00 Gy	79.20 %
	V 20.00 Gy	8.08 %
	V13.50 Gy	19.88 %
	D 1500.0 cm^3	8.55 Gy
**PRV_CaudaEquina**	D 0.1 cm^3	16.23 Gy
**PRV_SpinalCord**	D 0.1 cm^3	24.73 Gy
**Rectum**	D 0.1 cm^3	30.77 Gy
**Stomach**	D 0.1 cm^3	34.05 Gy
	D 10 cm^3	21.00 Gy
**Trachea**	D 0.1 cm^3	23.22 Gy

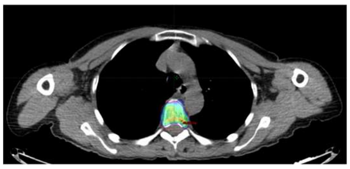

Esophagus

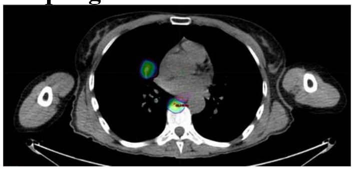

PRV_SpinalCord

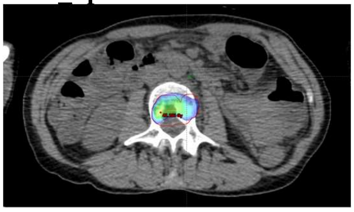

Stomach

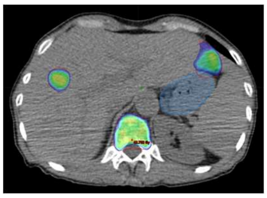

Patient_15

**N_isocenters = 2**	**Lesions = 15**	**Monitor Units: 6304.4**
**N_fields = 4 (2+2)**	**Under-dosed Lesions = 0**	

The Figure 10 below illustrate the spatial localization across the body of the PTV_all. In this case, no lesion was under-dosed to achieve an adequate plan. In Table 12 the dose-volume endpoints are reported.

Figure 10

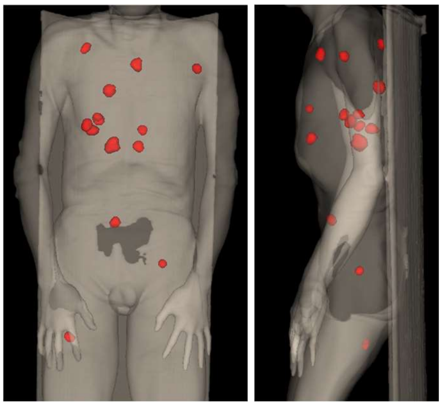

Table 12

**Dose Volume Endpoints**		
**Structure**	**Clinical Goal**	**Value**
**GTV_all**	V 110.0% > 95%	99.22 %
	123% < D 0.1 cm^3 < 126%	123.04 %
**PTV_all**	1.00 ≤ CI < 1.20	1.27
	V 100.0% > 95%	97.80 %
**Bladder**	D 15.0 cm^3	4.24 Gy

	D 0.1 cm^3	6.51 Gy
**Bowel**	D 0.1 cm^3	37.23 Gy
	D 20 cm^3	17.65 Gy
**BrachialPlexus_L**	D 3.0 cm^3	0.31 Gy
	D 0.1 cm^3	0.85 Gy
**BrachialPlexus_R**	D 3.0 cm^3	0.36 Gy
	D 0.1 cm^3	1.16 Gy
**BronchialTree**	D 5.0 cm^3	7.77 Gy
	D 0.1 cm^3	11.55 Gy
**Duodenum**	D 10 cm^3	2.03 Gy
	D 0.1 cm^3	4.06 Gy
**Esophagus**	D 0.1 cm^3	21.26 Gy
**FemurHead_L**	D 10 cm^3	28.18 Gy
**FemurHead_R**	D 10 cm^3	2.65 Gy
**GreatVessels**	D 0.1 cm^3	41.03 Gy
**Heart**	D 0.1 cm^3	18.27 Gy
	D 15.0 cm^3	14.46 Gy
**Kidneys – GTV_all**	D 200.0 cm^3	0.82 Gy
**Liver – GTV_all**	Dmean	13.23 Gy
	D 700 cm^3	12.45 Gy
**Lungs – GTV_all**	V 5.00 Gy	62.80 %
	V 20.00 Gy	3.55 %
	V13.50 Gy	11.46 %
	D 1500.0 cm^3	6.77 Gy
**PRV_CaudaEquina**	D 0.1 cm^3	2.84 Gy
**PRV_SpinalCord**	D 0.1 cm^3	20.03 Gy
**Rectum**	D 0.1 cm^3	5.30 Gy
**Stomach**	D 0.1 cm^3	21.07 Gy
	D 10 cm^3	12.64 Gy
**Trachea**	D 0.1 cm^3	16.73 Gy

Patient_16

**N_isocenters = 1**	**Lesions = 12**	**Monitor Units: 3763.4**
**N_fields = 3**	**Under-dosed Lesions = 2 (BronchialTree; Esophagus)**	

The Figure 11 below illustrate the spatial localization across the body of the PTV_all. In this case, no lesion was under-dosed to achieve an adequate plan. In Table 13 the dose-volume endpoints are reported.

Figure 11

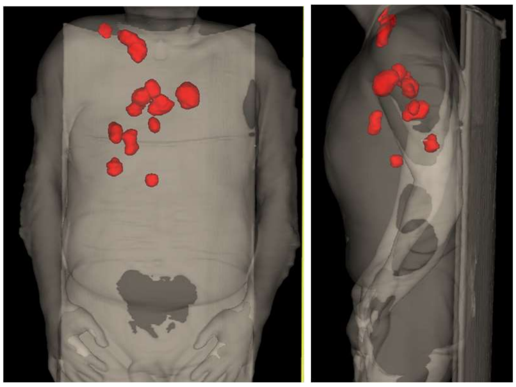

Table 13

**Dose Volume Endpoints**		
**Structure**	**Clinical Goal**	**Value**
**GTV_all**	V 110.0% > 95%	97.66 %
	123% < D 0.1 cm^3 < 126%	125.19 %
**PTV_all**	1.00 ≤ CI < 1.20	1.07
	V 100.0% > 95%	95.42 %
**Bladder**	D 15.0 cm^3	0.04 Gy

Below there are displayed the lesions for which a compromise was required; the 35 Gy isodose is highlighted):

BronchialTree & Esophagus

	D 0.1 cm^3	0.06 Gy
**Bowel**	D 0.1 cm^3	20.57 Gy
	D 20 cm^3	9.84 Gy
**BrachialPlexus_L**	D 3.0 cm^3	6.42 Gy
	D 0.1 cm^3	8.00 Gy
**BrachialPlexus_R**	D 3.0 cm^3	16.30 Gy
	D 0.1 cm^3	25.29 Gy
**BronchialTree**	D 5.0 cm^3	31.83 Gy
	D 0.1 cm^3	34.23 Gy
**Duodenum**	D 10 cm^3	16.52 Gy
	D 0.1 cm^3	23.89 Gy
**Esophagus**	D 0.1 cm^3	34.45 Gy
**FemurHead_L**	D 10 cm^3	0.00 Gy
**FemurHead_R**	D 10 cm^3	0.00 Gy
**GreatVessels**	D 0.1 cm^3	43.31 Gy
**Heart**	D 0.1 cm^3	33.18 Gy
	D 15.0 cm^3	20.28 Gy
**Kidneys – GTV_all**	D 200.0 cm^3	2.10 Gy
**Liver – GTV_all**	Dmean	13.03 Gy
	D 700 cm^3	13.67 Gy
**Lungs – GTV_all**	V 5.00 Gy	77.94 %
	V 20.00 Gy	8.58 %
	V13.50 Gy	29.38 %
	D 1500.0 cm^3	9.38 Gy
**PRV_CaudaEquina**	D 0.1 cm^3	0.54 Gy
**PRV_SpinalCord**	D 0.1 cm^3	23.27 Gy
**Rectum**	D 0.1 cm^3	0.02 Gy
**Stomach**	D 0.1 cm^3	7.62 Gy
	D 10 cm^3	6.25 Gy
**Trachea**	D 0.1 cm^3	35.88 Gy

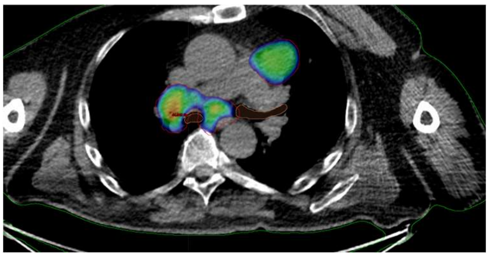

Patient_18

**N_isocenters = 2**	**Lesions = 15**	**Monitor Units: 3673.0**
**N_fields = 5 (3+2)**	**Under-dosed Lesions = 0**	

The Figure 12 below illustrate the spatial localization across the body of the PTV_all. In this case, no lesion was under-dosed to achieve an adequate plan. In Table 14 the dose-volume endpoints are reported.

Figure 12

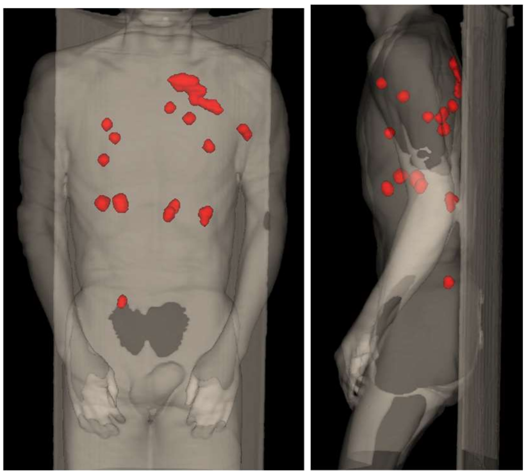

Table 14

**Dose Volume Endpoints**		
**Structure**	**Clinical Goal**	**Value**
**GTV_all**	V 110.0% > 95%	97.55 %

	123% < D 0.1 cm^3 < 126%	123.95 %
**PTV_all**	1.00 ≤ CI < 1.20	1.19
	V 100.0% > 95%	95.01 %
**Bladder**	D 15.0 cm^3	0.02 %
	D 0.1 cm^3	0.03 %
**Bowel**	D 0.1 cm^3	29.26 Gy
	D 20 cm^3	18.80 Gy
**BrachialPlexus_L**	D 3.0 cm^3	0.50 Gy
	D 0.1 cm^3	1.43 Gy
**BrachialPlexus_R**	D 3.0 cm^3	0.33 Gy
	D 0.1 cm^3	1.27 Gy
**BronchialTree**	D 5.0 cm^3	12.17 Gy
	D 0.1 cm^3	21.59 Gy
**Duodenum**	D 10 cm^3	2.65 Gy
	D 0.1 cm^3	29.33 Gy
**Esophagus**	D 0.1 cm^3	12.60 Gy
**FemurHead_L**	D 10 cm^3	0.02 Gy
**FemurHead_R**	D 10 cm^3	0.02 Gy
**GreatVessels**	D 0.1 cm^3	31.88 Gy
**Heart**	D 0.1 cm^3	16.49 Gy
	D 15.0 cm^3	11.86 Gy
**Kidneys – GTV_all**	D 200.0 cm^3	2.11 Gy
**Liver – GTV_all**	Dmean	9.18 Gy
	D 700 cm^3	7.38 Gy
**Lungs – GTV_all**	V 5.00 Gy	78.14 %
	V 20.00 Gy	5.03 %
	V13.50 Gy	14.53 %
	D 1500.0 cm^3	8.54 Gy
**PRV_CaudaEquina**	D 0.1 cm^3	1.28 Gy
**PRV_SpinalCord**	D 0.1 cm^3	15.52 Gy
**Rectum**	D 0.1 cm^3	0.05 Gy
**Stomach**	D 0.1 cm^3	33.57 Gy
	D 10 cm^3	20.58 Gy
**Trachea**	D 0.1 cm^3	12.18 Gy

Patient_20

**N_isocenters = 3**	**Lesions = 28**	**Monitor Units: 9409.5**
**N_fields = 8 (2+4+2)**	**Under-dosed Lesions = 2 (1 PRV_SpinalCord; 1 Stomach)**	

The Figure 13 below illustrate the spatial localization across the body of the PTV_all. In this case, no lesion was under-dosed to achieve an adequate plan. In Table 15 the dose-volume endpoints are reported.

Figure 13

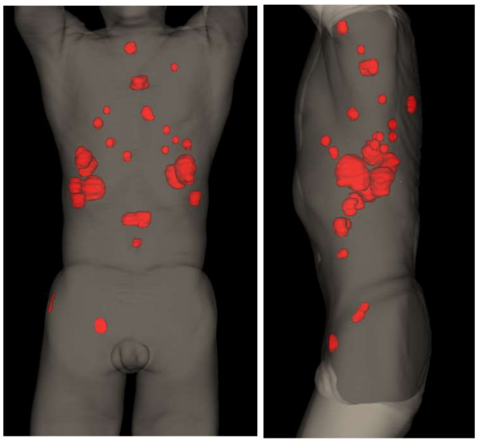

Table 15

Dose Volume Endpoints		
**Structure**	**Clinical Goal**	**Value**
**GTV_all**	V 110.0% > 95%	95.69 %
	123% < D 0.1 cm^3 < 126%	125.65 %
**PTV_all**	1.00 ≤ CI < 1.20	1.08

Below there are displayed the lesions for which a compromise was required; the 35 Gy isodose is highlighted):

PRV_SpinalCord

	V 100.0% > 95%	95.01 %
Bladder	D 15.0 cm^3	4.37 Gy
	D 0.1 cm^3	6.55 Gy
Bowel	D 0.1 cm^3	36.88 Gy
	D 20 cm^3	25.71 Gy
BrachialPlexus_L	D 3.0 cm^3	5.56 Gy
	D 0.1 cm^3	8.42 Gy
BrachialPlexus_R	D 3.0 cm^3	2.36 Gy
	D 0.1 cm^3	19.83 Gy
BronchialTree	D 5.0 cm^3	8.71 Gy
	D 0.1 cm^3	15.44 Gy
Duodenum	D 10 cm^3	19.60 Gy
	D 0.1 cm^3	33.82 Gy
Esophagus	D 0.1 cm^3	32.96 Gy
FemurHead_L	D 10 cm^3	2.42 Gy
FemurHead_R	D 10 cm^3	8.83 Gy
GreatVessels	D 0.1 cm^3	38.45 Gy
Heart	D 0.1 cm^3	34.05 Gy
	D 15.0 cm^3	22.50 Gy
Kidneys – GTV_all	D 200.0 cm^3	9.68 Gy
Liver – GTV_all	Dmean	15.57 Gy
	D 700 cm^3	14.86 Gy
Lungs – GTV_all	V 5.00 Gy	79.24 %
	V 20.00 Gy	11.92 %
	V13.50 Gy	28.12 %
	D 1500.0 cm^3	10.51 Gy
PRV_CaudaEquina	D 0.1 cm^3	3.68 Gy
PRV_SpinalCord	D 0.1 cm^3	26.45 Gy
Rectum	D 0.1 cm^3	5.45 Gy
Stomach	D 0.1 cm^3	31.34 Gy
	D 10 cm^3	22.92 Gy
Trachea	D 0.1 cm^3	35.07 Gy

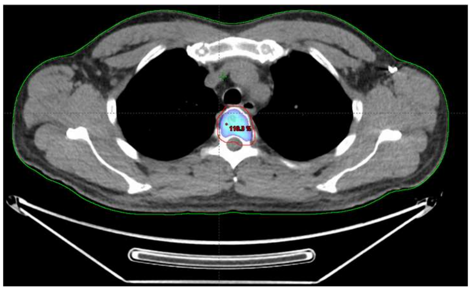

Stomach

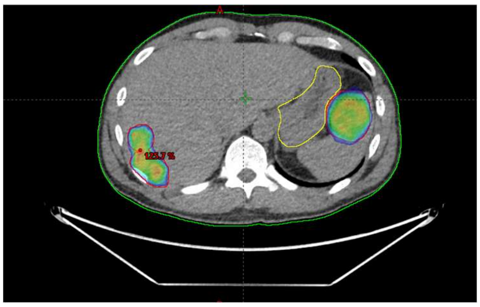

Patient_21

N_isocenters = 3	Lesions = 22	Monitor Units: 12728.5
N_fields = 8 (4+2+2)	Under-dosed Lesions = 2 (2 Bowel)	

The Figure 14 below illustrate the spatial localization across the body of the PTV_all. In this case, no lesion was under-dosed to achieve an adequate plan. In Table 16 the dose-volume endpoints are reported.

Figure 14

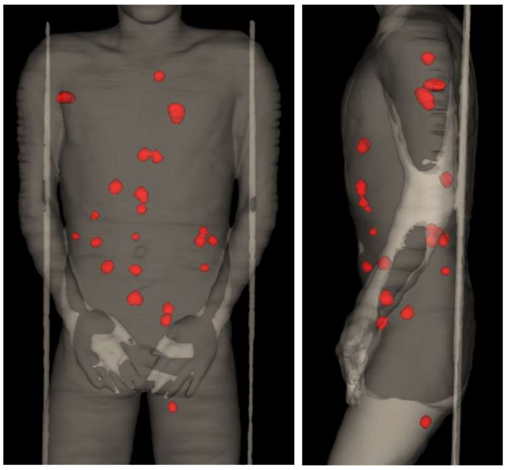

Table 16

Dose Volume Endpoints		
Structure	Clinical Goal	Value
GTV_all	V 110.0% > 95%	95.02 %
	123% < D 0.1 cm^3 < 126%	123.01 %

Below there are displayed the lesions for which a compromise was required; the 35 Gy isodose is highlighted:

**PTV_all**	1.00 ≤ CI < 1.20	1.19
	V 100.0% > 95%	95.02 %
**Bladder**	D 15.0 cm^3	1.19 Gy
	D 0.1 cm^3	1.75 Gy
**Bowel**	D 0.1 cm^3	37.32 Gy
	D 20 cm^3	28.24 Gy
**BrachialPlexus_L**	D 3.0 cm^3	0.28 Gy
	D 0.1 cm^3	0.48 Gy
**BrachialPlexus_R**	D 3.0 cm^3	0.25 Gy
	D 0.1 cm^3	0.44 Gy
**BronchialTree**	D 5.0 cm^3	18.35 Gy
	D 0.1 cm^3	36.29 Gy
**Duodenum**	D 10 cm^3	11.95 Gy
	D 0.1 cm^3	22.18 Gy
**Esophagus**	D 0.1 cm^3	22.41 Gy
**FemurHead_L**	D 10 cm^3	1.87 Gy
**FemurHead_R**	D 10 cm^3	1.14 Gy
**GreatVessels**	D 0.1 cm^3	30.59 Gy
**Heart**	D 0.1 cm^3	36.59 Gy
	D 15.0 cm^3	24.07 Gy
**Kidneys – GTV_all**	D 200.0 cm^3	4.97 Gy
**Liver – GTV_all**	Dmean	8.89 Gy
	D 700 cm^3	8.52 Gy
**Lungs – GTV_all**	V 5.00 Gy	43.28 %
	V 20.00 Gy	1.54 %
	V13.50 Gy	4.38 %
	D 1500.0 cm^3	6.65 Gy
**PRV_CaudaEquina**	D 0.1 cm^3	11.31 Gy
**PRV_SpinalCord**	D 0.1 cm^3	23.45 Gy
**Rectum**	D 0.1 cm^3	15.07 Gy
**Stomach**	D 0.1 cm^3	16.96 Gy
	D 10 cm^3	12.98 Gy
**Trachea**	D 0.1 cm^3	14.67 Gy

Bowel

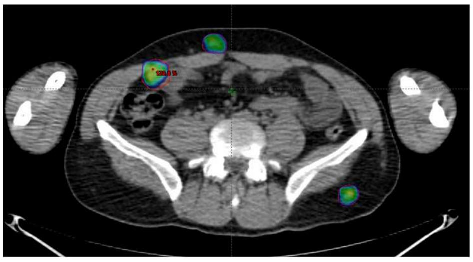

Bowel

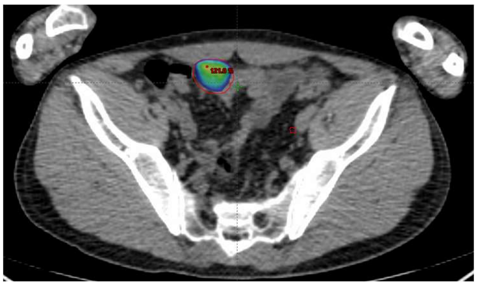

Patient_22

**N_isocenters = 3**	**Lesions = 15**	**Monito Units: 8654.6**
**N_fields = 6 (2+2+2)**	**Under-dosed Lesions = 1 (1 BronchialTree)**	

The Figure 15 below illustrate the spatial localization across the body of the PTV_all. In this case, no lesion was under-dosed to achieve an adequate plan. In Table 17 the dose-volume endpoints are reported.

Figure 15

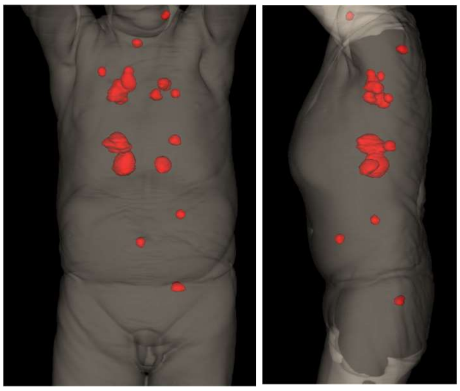

Table 17

**Dose Volume Endpoints**		
**Structure**	**Clinical Goal**	**Value**
**GTV_all**	V 110.0% > 95%	97.84 %
	123% < D 0.1 cm^3 < 126%	123.01 %
**PTV_all**	1.00 ≤ CI < 1.20	1.02
	V 100.0% > 95%	95.60 %

Below there is displayed the lesion for which a compromise was required; the 35 Gy isodose is highlighted:

BronchialTree

**Bladder**	D 15.0 cm^3	5.29 Gy
	D 0.1 cm^3	8.64 Gy
**Bowel**	D 0.1 cm^3	37.96 Gy
	D 20 cm^3	16.10 Gy
**BrachialPlexus_L**	D 3.0 cm^3	1.70 Gy
	D 0.1 cm^3	4.04 Gy
**BrachialPlexus_R**	D 3.0 cm^3	2.16 Gy
	D 0.1 cm^3	6.55 Gy
**BronchialTree**	D 5.0 cm^3	30.51 Gy
	D 0.1 cm^3	34.81 Gy
**Duodenum**	D 10 cm^3	17025 Gy
	D 0.1 cm^3	26.17 Gy
**Esophagus**	D 0.1 cm^3	24.45 Gy
**FemurHead_L**	D 10 cm^3	7.92 Gy
**FemurHead_R**	D 10 cm^3	1.56 Gy
**GreatVessels**	D 0.1 cm^3	41.96 Gy
**Heart**	D 0.1 cm^3	25.54 Gy
	D 15.0 cm^3	13.35 Gy
**Kidneys – GTV_all**	D 200.0 cm^3	1.79 Gy
**Liver – GTV_all**	Dmean	13.94 Gy
	D 700 cm^3	13.30 Gy
**Lungs – GTV_all**	V 5.00 Gy	63.74 %
	V 20.00 Gy	8.97 %
	V13.50 Gy	27.24 %
	D 1500.0 cm^3	10.46 Gy
**PRV_CaudaEquina**	D 0.1 cm^3	6.21 Gy
**PRV_SpinalCord**	D 0.1 cm^3	23.58 Gy
**Rectum**	D 0.1 cm^3	7.80 Gy
**Stomach**	D 0.1 cm^3	24.04 Gy
	D 10 cm^3	20.15 Gy
**Trachea**	D 0.1 cm^3	34.51 Gy

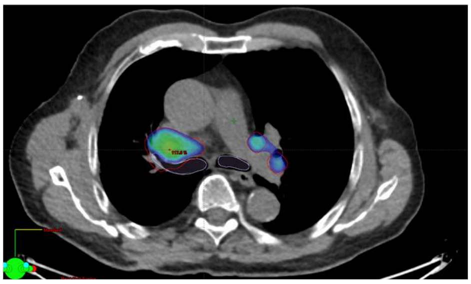

Patient_23

**N_isocenters = 2**	**Lesions = 23**	**Monitor Units: 7724.6**
**N_fields = 5 (3+2)**	**Under-dosed Lesions = 2 (1 Trachea; 1 Stomach)**	

The Figure 16 below illustrate the spatial localization across the body of the PTV_all. In this case, no lesion was under-dosed to achieve an adequate plan. In Table 18 the dose-volume endpoints are reported.

Figure 16

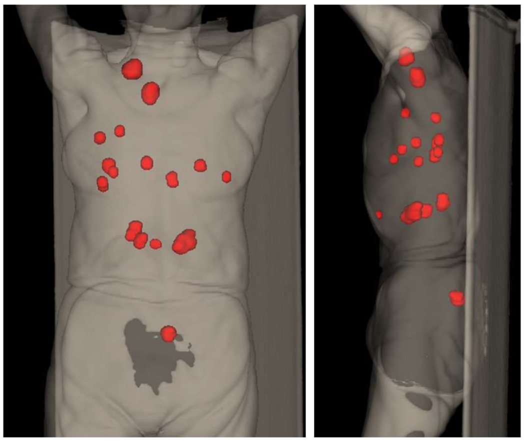

Table 18

Dose Volume Endpoints		
**Structure**	**Clinical Goal**	**Value**
**GTV_all**	V 110.0% > 95%	97.61 %
	123% < D 0.1 cm^3 < 126%	123.01 %

Below there is displayed the lesion for which a compromise was required; the 35 Gy isodose is highlighted:

**PTV_all**	1.00 ≤ CI < 1.20	1.19
	V 100.0% > 95%	96.15 %
**Bladder**	D 15.0 cm^3	0.18 Gy
	D 0.1 cm^3	0.31 Gy
**Bowel**	D 0.1 cm^3	35.83 Gy
	D 20 cm^3	20.23 Gy
**BrachialPlexus_L**	D 3.0 cm^3	0.88 Gy
	D 0.1 cm^3	4.28 Gy
**BrachialPlexus_R**	D 3.0 cm^3	1.25 Gy
	D 0.1 cm^3	25.72 Gy
**BronchialTree**	D 5.0 cm^3	10.29 Gy
	D 0.1 cm^3	14.77 Gy
**Duodenum**	D 10 cm^3	19.45 Gy
	D 0.1 cm^3	33.28 Gy
**Esophagus**	D 0.1 cm^3	33.40 Gy
**FemurHead_L**	D 10 cm^3	0.12 Gy
**FemurHead_R**	D 10 cm^3	0.14 Gy
**GreatVessels**	D 0.1 cm^3	33.84 Gy
**Heart**	D 0.1 cm^3	32.90 Gy
	D 15.0 cm^3	21.86 Gy
**Kidneys – GTV_all**	D 200.0 cm^3	9.03
**Liver – GTV_all**	Dmean	11.26 Gy
	D 700 cm^3	4.57 Gy
**Lungs – GTV_all**	V 5.00 Gy	69.93 %
	V 20.00 Gy	12.58 %
	V13.50 Gy	29.27 %
	D 1500.0 cm^3	7.92 Gy
**PRV_CaudaEquina**	D 0.1 cm^3	25.88 Gy
**PRV_SpinalCord**	D 0.1 cm^3	23.75 Gy
**Rectum**	D 0.1 cm^3	0.15 Gy
**Stomach**	D 0.1 cm^3	31.75 Gy
	D 10 cm^3	22.86 Gy
**Trachea**	D 0.1 cm^3	36.84 Gy

Trachea

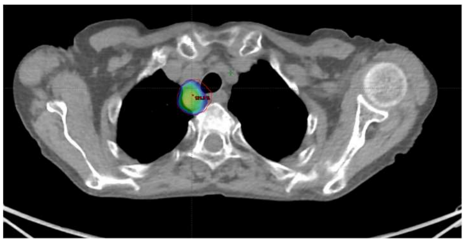

Stomach

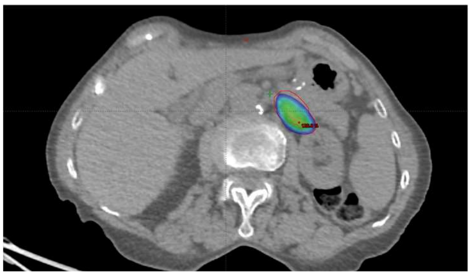

f. Constrains Violation in Rejected Plans

Out of 23 plans, 7 were rejected due to constraints violation (i.e., 35 Gy in 5 fractions at 80% of the isodose line).

In the table below (Table 19) are shown the dose limiting constraints (left column) and in many cases, they precluded the administration of the prescribed dose (right column).

Table 19

**Clinical Goal**	**N**
BronchialTree_D0.1 cm^3	1
Heart_ D0.1 cm^3	1
Heart_ D15 cm^3	1
FemurHead_R_ D 10 cm^3	1
Lungs – GTV_all_V5.00 Gy	4
Lungs – GTV_all_V20.00 Gy	4
Lungs – GTV_all_V13.50 Gy	4

Lung – GTV_all_ D1500.0 cm^3	4
Liver-GTV_all_Dmean	1
Liver-GTV_all_D700.0 cm^3	2
PRV_SpinalCord_ D0.1 cm^3	1
PRV_CaudaEquina_ D0.1 cm^3	1
Stomach_ D0.1 cm^3	3

In the next pages, the rejected plans are reported in detail, highlighting their dose-volume endpoints with pictures of the spatial configuration of their lesion.

**Patient_6**

**N_isocenters = 2**	**Lesions = 37**
**N_fields = 7 (5+2)**	**Violated Constrains = Lungs-GTV_all**

17a. **17b.** **17c.** **17d.**

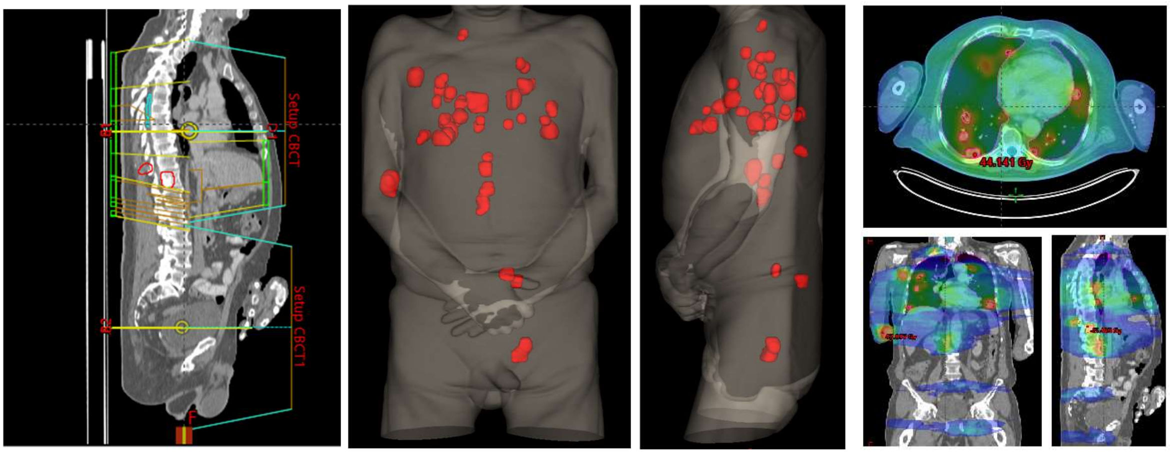

The figure above illustrates the number of isocenters (Fig.17a) used and the lesions spread in the body in the coronal (Fig 17b) and sagittal (Fig 17c) view by highlighting the PTV_all. Fig 17d shows the dose color wash in the three dimensions. For the legend see below on the right.

The Table 20 below on the left reports the dose volume endpoints, indicating which the clinical goals were violated.

Dose Volume Endpoints			
**Structure**	**Clinical Goal**	**Value**	**Statutus**
**GTV_all**	V 110.0% > 95%	95.29%	Meet
	123% < D 0.1 cm^3 < 126%	125.68%	Meet
**PTV_all**	1.00 ≤ CI < 1.20	1.10	Meet
	V 100.0% > 95%	95.01%	Meet
**Bladder**	D 15.0 cm^3	2.17 Gy	Meet
	D 0.1 cm^3	3.31 Gy	Meet
**Bowel**	D 0.1 cm^3	13.29 Gy	Meet
	D 20 cm^3	10.31 Gy	Meet
**BrachialPlexus_L**	D 3.0 cm^3	2.47 Gy	Meet
	D 0.1 cm^3	3.32 Gy	Meet
**BrachialPlexus_R**	D 3.0 cm^3	18.33 Gy	Meet
	D 0.1 cm^3	29.39 Gy	Meet
**BronchialTree**	D 5.0 cm^3	19.81 Gy	Meet
	D 0.1 cm^3	34.05 Gy	Meet
**Duodenum**	D 10 cm^3	12.81 Gy	Meet
	D 0.1 cm^3	14.84 Gy	Meet
**Esophagus**	D 0.1 cm^3	29.15 Gy	Meet
**FemurHead_L**	D 10 cm^3	7.15 Gy	Meet
**FemurHead_R**	D 10 cm^3	5.76 Gy	Meet
**GreatVessels**	D 0.1 cm^3	41.51 Gy	Meet
**Heart**	D 0.1 cm^3	37.14 Gy	Meet
	D 15.0 cm^3	28.40 Gy	Meet
**Kidneys – GTV_all**	D 200.0 cm^3	6.54 Gy	Meet
**Liver – GTV_all**	Dmean	9.06 Gy	Meet
	D 700 cm^3	9.52 Gy	Meet
**Lungs – GTV_all**	V 5.00 Gy	97.51 %	Violated
	V 20.00 Gy	54.36 %	Violated
	V13.50 Gy	80.35 %	Violated
	D 1500.0 cm^3	24.47 %	Violated
**PRV_CaudaEquina**	D 0.1 cm^3	22.70 Gy	Meet
**PRV_SpinalCord**	D 0.1 cm^3	22.94 Gy	Meet
**Rectum**	D 0.1 cm^3	13.86 Gy	Meet
**Stomach**	D 0.1 cm^3	12.36 Gy	Meet
	D 10 cm^3	10.60 Gy	Meet
**Trachea**	D 0.1 cm^3	13.94 Gy	Meet

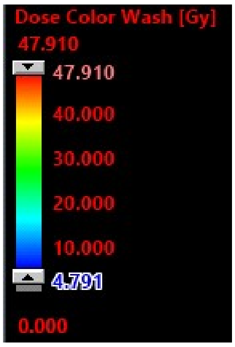

Above is reported the dose color wash of the figure 17d.

Patient_8

**N_isocenters = 1**	**Lesions = 26**
**N_fields = 3**	**Violated Constrains = Lungs-GTV_all ; Stomach**

18a. **18b.** **18c.** **18d.**

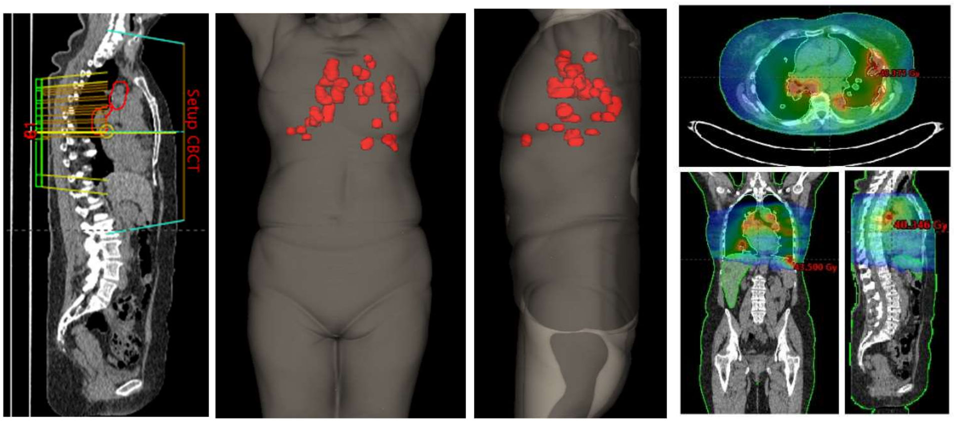

The figure above illustrates the number of isocenters (Fig.18a) used and the lesions spread in the body in the coronal (Fig 18b) and sagittal (Fig 18c) view by highlighting the PTV_all. Fig 18d shows he dose color wash in the three dimensions. For the legend see below on the right.

The Table 21 below on the left reports the dose volume endpoints, indicating which the clinical goals were violated.

Dose Volume Endpoints			
**Structure**	**Clinical Goal**	**Value**	**Statutus**
**GTV_all**	V 110.0% > 95%	97.06 %	Meet
	123% < D 0.1 cm^3 < 126%	124.29 %	Meet
**PTV_all**	1.00 ≤ CI < 1.20	1.18	Meet
	V 100.0% > 95%	95.01 %	Meet
**Bladder**	D 15.0 cm^3	0.00 Gy	Meet
	D 0.1 cm^3	0.00 Gy	Meet
**Bowel**	D 0.1 cm^3	18.72 Gy	Meet
	D 20 cm^3	3.03 Gy	Meet
**BrachialPlexus_L**	D 3.0 cm^3	0.56 Gy	Meet
	D 0.1 cm^3	1.02 Gy	Meet
**BrachialPlexus_R**	D 3.0 cm^3	0.51 Gy	Meet
	D 0.1 cm^3	0.88 Gy	Meet
**BronchialTree**	D 5.0 cm^3	31.76 Gy	Meet
	D 0.1 cm^3	35.28 Gy	Meet
**Duodenum**	D 10 cm^3	0.79 Gy	Meet
	D 0.1 cm^3	1.32 Gy	Meet
**Esophagus**	D 0.1 cm^3	33.75 Gy	Meet
**FemurHead_L**	D 10 cm^3	0.00 Gy	Meet
**FemurHead_R**	D 10 cm^3	0.00 Gy	Meet
**GreatVessels**	D 0.1 cm^3	41.63 Gy	Meet
**Heart**	D 0.1 cm^3	37.70 Gy	Meet
	D 15.0 cm^3	29.64 Gy	Meet
**Kidneys – GTV_all**	D 200.0 cm^3	0.50 Gy	Meet
**Liver – GTV_all**	Dmean	5.15 Gy	Meet
	D 700 cm^3	1.29 Gy	Meet
**Lungs – GTV_all**	V 5.00 Gy	95.15 %	Violated
	V 20.00 Gy	44.48 %	Violated
	V13.50 Gy	78.89 %	Violated
	D 1500.0 cm^3	18.33 Gy	Violated
**PRV_CaudaEquina**	D 0.1 cm^3	0.61 Gy	Meet
**PRV_SpinalCord**	D 0.1 cm^3	23.94 Gy	Meet
**Rectum**	D 0.1 cm^3	0.00 Gy	Meet
**Stomach**	D 0.1 cm^3	33.22 Gy	Violated
	D 10 cm^3	18.33 Gy	Meet
**Trachea**	D 0.1 cm^3	37.81 Gy	Meet

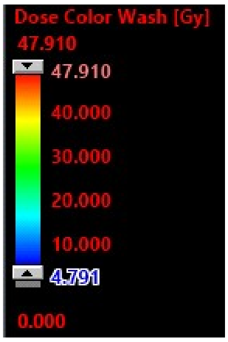

Above is reported the dose color wash of the figure 18d.

Patient_9

**N_isocenters = 2**	**Lesions = 11**
**N_fields = 5 (3+2)**	**Violated Constrains = Lungs-GTV_all**

19a. **19b.** **19c.** **19d.**

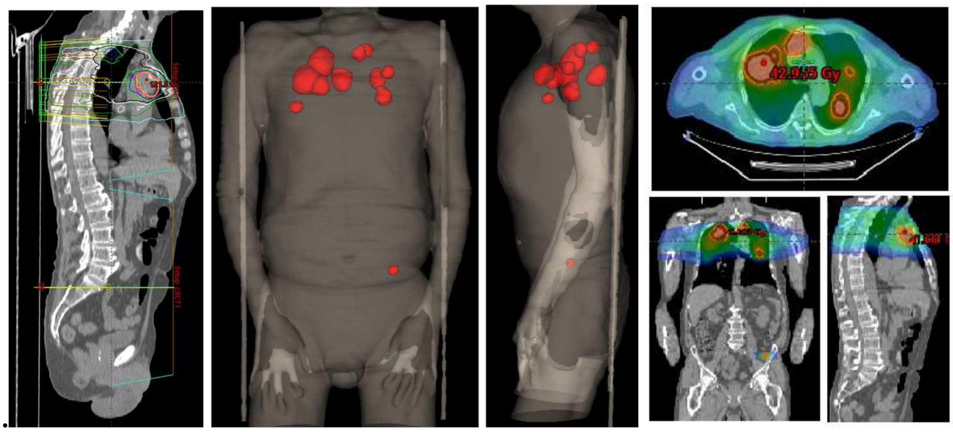

The figure above illustrates the number of isocenters (Fig.19a) used and the lesions spread in the body in the coronal (Fig 19b) and sagittal (Fig 19c) view by highlighting the PTV_all. Fig 19d shows the isodose levels in the dose color wash. For legend see below on the right.

The Table 22 below on the left reports the dose volume endpoints, indicating which the clinical goals were violated.

Dose Volume Endpoints			
**Structure**	**Clinical Goal**	**Value**	**Statutus**
**GTV_all**	V 110.0% > 95%	99.03 %	Meet
	123% < D 0.1 cm^3 < 126%	125.11 %	Meet
**PTV_all**	1.00 ≤ CI < 1.20	1.01	Meet
	V 100.0% > 95%	95.01 %	Meet
**Bladder**	D 15.0 cm^3	0.02 Gy	Meet
	D 0.1 cm^3	0.03 Gy	Meet
**Bowel**	D 0.1 cm^3	23.95 Gy	Meet
	D 20 cm^3	9.24 Gy	Meet
**BrachialPlexus_L**	D 3.0 cm^3	1.31 Gy	Meet
	D 0.1 cm^3	7.80 Gy	Meet
**BrachialPlexus_R**	D 3.0 cm^3	3.70 Gy	Meet
	D 0.1 cm^3	17.77 Gy	Meet
**BronchialTree**	D 5.0 cm^3	21.40 Gy	Meet
	D 0.1 cm^3	26.06 Gy	Meet
**Duodenum**	D 10 cm^3	0.18 Gy	Meet
	D 0.1 cm^3	0.20 Gy	Meet
**Esophagus**	D 0.1 cm^3	28.15 Gy	Meet
**FemurHead_L**	D 10 cm^3	0.05 Gy	Meet
**FemurHead_R**	D 10 cm^3	0.01 Gy	Meet
**GreatVessels**	D 0.1 cm^3	40.47 Gy	Meet
**Heart**	D 0.1 cm^3	18.44 Gy	Meet
	D 15.0 cm^3	13.74 Gy	Meet
**Kidneys – GTV_all**	D 200.0 cm^3	0.11 Gy	Meet
**Liver – GTV_all**	Dmean	0.27 Gy	Meet
	D 700 cm^3	0.24 Gy	Meet
**Lungs – GTV_all**	V 5.00 Gy	56.14 %	Meet
	V 20.00 Gy	30.45 %	Violated
	V13.50 Gy	43.49 %	Violated
	D 1500.0 cm^3	18.88 Gy	Violated
**PRV_CaudaEquina**	D 0.1 cm^3	0.08 Gy	Meet
**PRV_SpinalCord**	D 0.1 cm^3	22.62 Gy	Meet
**Rectum**	D 0.1 cm^3	0.03 Gy	Meet
**Stomach**	D 0.1 cm^3	0.50 Gy	Meet
	D 10 cm^3	0.44 Gy	Meet
**Trachea**	D 0.1 cm^3	29.99 Gy	Meet

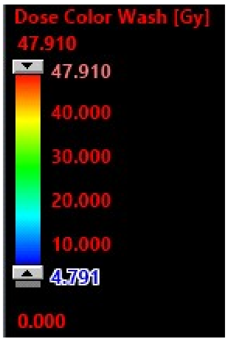

Above is reported the dose color wash of the figure 19d.

Patient_12

**N_isocenters = 3**	**Lesions = 18**
**N_fields = 8 (3+2+3)**	**Violated Constrains = Lungs-GTV_all ; Heart ; Bronchial Tree**

20a. **20b.** **20c.** **20d.**

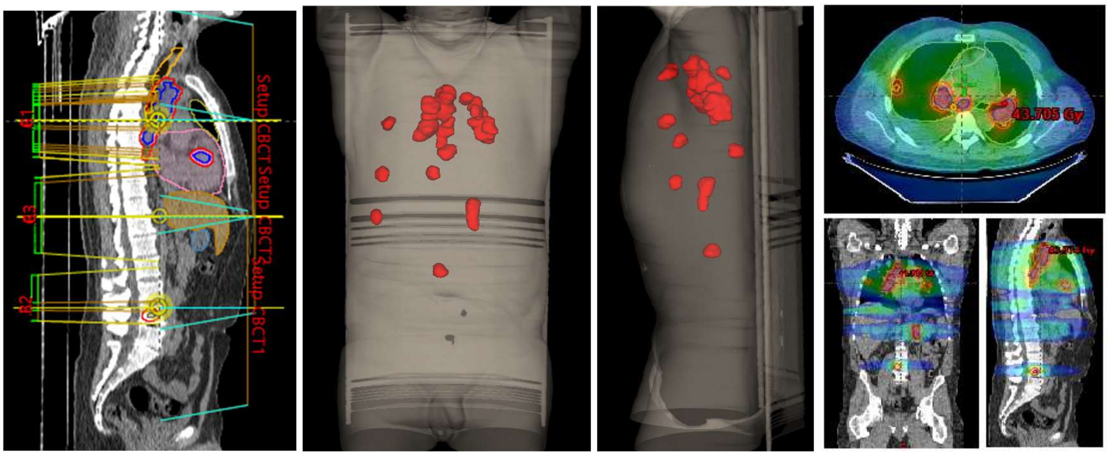

The figure above illustrates the number of isocenters (Fig.20a) used and the lesions spread in the body in the coronal (Fig 20b) and sagittal (Fig 20c) view by highlighting the PTV_all. Fig 20d shows the dose color wash in the three dimensions. For the legend see below on the right.

The table 23 below on the left reports the dose volume endpoints, indicating which the clinical goals were violated.

Dose Volume Endpoints			
**Structure**	**Clinical Goal**	**Value**	**Statutus**
**GTV_all**	V 110.0% > 95%	95.01 %	Meet
	123% < D 0.1 cm^3 < 126%	124.87%	Meet
**PTV_all**	1.00 ≤ CI < 1.20	1.18	Meet
	V 100.0% > 95%	95.40 %	Meet
**Bladder**	D 15.0 cm^3	0.04 Gy	Meet
	D 0.1 cm^3	0.05 Gy	Meet
**Bowel**	D 0.1 cm^3	27.55 Gy	Meet
	D 20 cm^3	15.99 Gy	Meet
**BrachialPlexus_L**	D 3.0 cm^3	1.29 Gy	Meet
	D 0.1 cm^3	1.86 Gy	Meet
**BrachialPlexus_R**	D 3.0 cm^3	1.07 Gy	Meet
	D 0.1 cm^3	1.60 Gy	Meet
**BronchialTree**	D 5.0 cm^3	32.72 Gy	Violated
	D 0.1 cm^3	35.74 Gy	Meet
**Duodenum**	D 10 cm^3	2.92 Gy	Meet
	D 0.1 cm^3	15.53 Gy	Meet
**Esophagus**	D 0.1 cm^3	33.93 Gy	Meet
**FemurHead_L**	D 10 cm^3	0.03 Gy	Meet
**FemurHead_R**	D 10 cm^3	0.03 Gy	Meet
**GreatVessels**	D 0.1 cm^3	43.17 Gy	Meet
**Heart**	D 0.1 cm^3	40.76 Gy	Violated
	D 15.0 cm^3	34.14 Gy	Violated
**Kidneys – GTV_all**	D 200.0 cm^3	6.70 Gy	Meet
**Liver – GTV_all**	Dmean	9.18 Gy	Meet
	D 700 cm^3	11.00 Gy	Meet
**Lungs – GTV_all**	V 5.00 Gy	92.96 %	Violated
	V 20.00 Gy	36.47 %	Violated
	V13.50 Gy	59.69 %	Violated
	D 1500.0 cm^3	15.63 Gy	Violated
**PRV_CaudaEquina**	D 0.1 cm^3	28.25 Gy	Meet
**PRV_SpinalCord**	D 0.1 cm^3	23.75 Gy	Meet
**Rectum**	D 0.1 cm^3	0.07 Gy	Meet
**Stomach**	D 0.1 cm^3	16.53 Gy	Meet
	D 10 cm^3	20.51 Gy	Meet
**Trachea**	D 0.1 cm^3	37.73 Gy	Meet

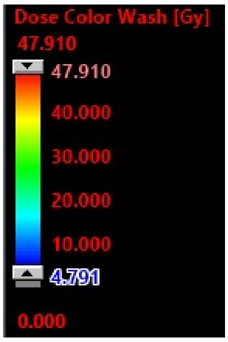

Above is reported the dose color wash of the figure 20d.

Patient_14

**N_isocenters = 3**	**Lesions = 24**
**N_fields = 6 (2+2+2)**	**Violated Constrains = Stomach; Liver-GTV_all**

21a. **21b.** **21c.** **21d.**

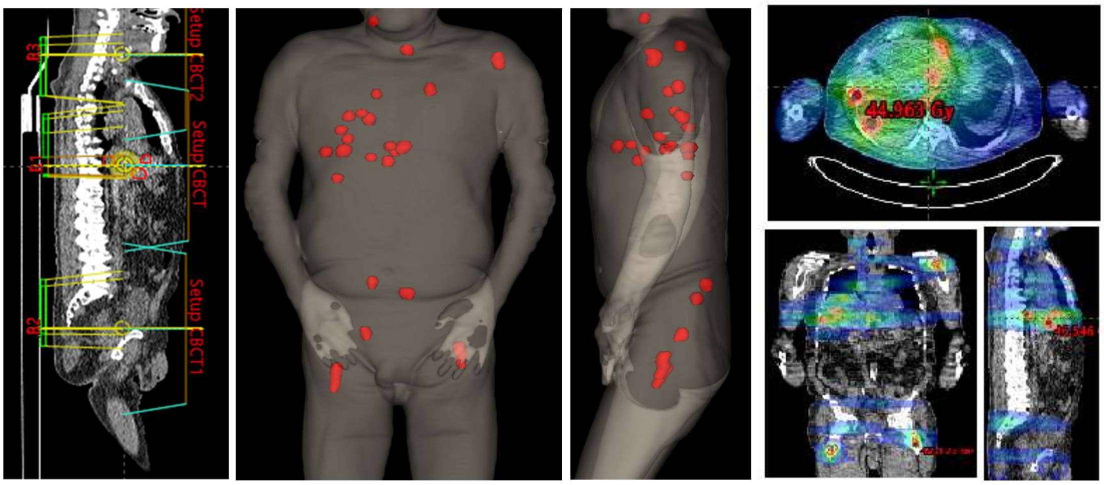

The figure above illustrates the number of isocenters (Fig.21a) used and the lesions spread in the body in the coronal (Fig 21b) and sagittal (Fig 21c) view by highlighting the PTV_all. Fig 21d shows the dose color wash the three dimensions. For the

legend see below on the right. The Table 24 below on the left reports the dose volume endpoints, indicating which the clinical goals were violated.

Dose Volume Endpoints			
**Structure**	**Clinical Goal**	**Value**	**Statutus**
**GTV_all**	V 110.0% > 95%	97.93 %	Meet
	123% < D 0.1 cm^3 < 126%	123.03 %	Meet
**PTV_all**	1.00 ≤ CI < 1.20	1.29	Meet
	V 100.0% > 95%	96.90 %	Meet
**Bladder**	D 15.0 cm^3	8.54 Gy	Meet
	D 0.1 cm^3	11.50 Gy	Meet
**Bowel**	D 0.1 cm^3	29.09 Gy	Meet
	D 20 cm^3	14.86 Gy	Meet
**BrachialPlexus_L**	D 3.0 cm^3	5.22 Gy	Meet
	D 0.1 cm^3	15.47 Gy	Meet
**BrachialPlexus_R**	D 3.0 cm^3	7.38 Gy	Meet
	D 0.1 cm^3	10.67 Gy	Meet
**BronchialTree**	D 5.0 cm^3	10.18 Gy	Meet
	D 0.1 cm^3	19.93 Gy	Meet
**Duodenum**	D 10 cm^3	6.67 Gy	Meet
	D 0.1 cm^3	20.98 Gy	Meet
**Esophagus**	D 0.1 cm^3	26.04 Gy	Meet
**FemurHead_L**	D 10 cm^3	40.53 Gy*	Violated
**FemurHead_R**	D 10 cm^3	30.00 Gy	Meet
**GreatVessels**	D 0.1 cm^3	38.98 Gy	Meet
**Heart**	D 0.1 cm^3	37.17 Gy	Meet
	D 15.0 cm^3	22.69 Gy	Meet
**Kidneys – GTV_all**	D 200.0 cm^3	0.48 Gy	Meet
**Liver – GTV_all**	Dmean	17.62 Gy	Meet
	D 700 cm^3	17.23 Gy	Violated
**Lungs – GTV_all**	V 5.00 Gy	78.49 %	Meet
	V 20.00 Gy	3.01 %	Meet
	V13.50 Gy	10.90 %	Meet
	D 1500.0 cm^3	5.60 Gy	Meet
**PRV_CaudaEquina**	D 0.1 cm^3	16.89 Gy	Meet
**PRV_SpinalCord**	D 0.1 cm^3	27.04 Gy	Meet
**Rectum**	D 0.1 cm^3	11.88 Gy	Meet
**Stomach**	D 0.1 cm^3	33.36 Gy	Violated
	D 10 cm^3	16.08 Gy	Meet
**Trachea**	D 0.1 cm^3	18.30 Gy	Meet

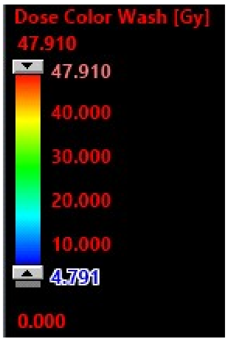

Above is reported the dose color wash of the figure 21d.

Note: *The patient has a target lesion into the FemorHead_L

Patient_17

**N_isocenters = 2**	**Lesions = 51**
**N_fields = 6 (4+2)**	**Violated Constrains = Stomach; Liver-GTV_all**

22a. **22b.** **22c.** **22d.**

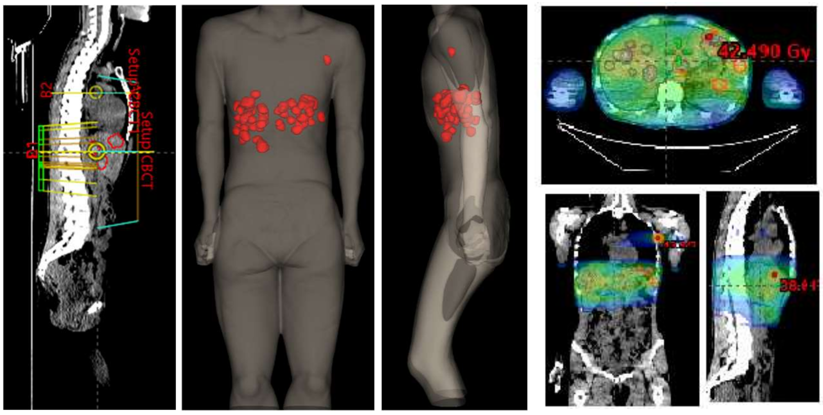

The figure above illustrates the number of isocenters (Fig.22a) used and the lesions spread in the body in the coronal (Fig 22b) and sagittal (Fig 22c) view by highlighting the PTV_all. Fig 22d shows the isodose levels in the three dimensions. For the isodose levels legend see below on the right.

The table 25 below on the left reports the dose volume endpoints, indicating which the clinical goals were violated.

Dose Volume Endpoints			
**Structure**	**Clinical Goal**	**Value**	**Statutus**
**GTV_all**	V 110.0% > 95%	98.81 %	Meet
	123% < D 0.1 cm^3 < 126%	124.63 %	Meet
**PTV_all**	1.00 ≤ CI < 1.20	1.22	Meet
	V 100.0% > 95%	95.01 %	Meet
**Bladder**	D 15.0 cm^3	0.00 Gy	Meet
	D 0.1 cm^3	0.04 Gy	Meet
**Bowel**	D 0.1 cm^3	19.49 Gy	Meet
	D 20 cm^3	9.11 Gy	Meet
**BrachialPlexus_L**	D 3.0 cm^3	0.08 Gy	Meet
	D 0.1 cm^3	0.21 Gy	Meet
**BrachialPlexus_R**	D 3.0 cm^3	0.02 Gy	Meet
	D 0.1 cm^3	0.17 Gy	Meet
**BronchialTree**	D 5.0 cm^3	1.69 Gy	Meet
	D 0.1 cm^3	2.59 Gy	Meet
**Duodenum**	D 10 cm^3	1.64 Gy	Meet
	D 0.1 cm^3	20.23 Gy	Meet
**Esophagus**	D 0.1 cm^3	33.51 Gy	Meet
**FemurHead_L**	D 10 cm^3	0.00 Gy	Meet
**FemurHead_R**	D 10 cm^3	0.00 Gy	Meet
**GreatVessels**	D 0.1 cm^3	37.83 Gy	Meet
**Heart**	D 0.1 cm^3	37.13 Gy	Meet
	D 15.0 cm^3	25.10 Gy	Meet
**Kidneys – GTV_all**	D 200.0 cm^3	3.65 Gy	Meet
**Liver – GTV_all**	Dmean	28.26 Gy	Violated
	D 700 cm^3	32.28 Gy	Violated
**Lungs – GTV_all**	V 5.00 Gy	20.15 %	Meet
	V 20.00 Gy	5.79 %	Meet
	V13.50 Gy	11.05 %	Meet
	D 1500.0 cm^3	1.50 Gy	Meet
**PRV_CaudaEquina**	D 0.1 cm^3	1.17 Gy	Meet
**PRV_SpinalCord**	D 0.1 cm^3	21.88 Gy	Meet
**Rectum**	D 0.1 cm^3	0.04 Gy	Meet
**Stomach**	D 0.1 cm^3	32.73 Gy	Violated
	D 10 cm^3	22.02 Gy	Meet
**Trachea**	D 0.1 cm^3	0.67 Gy	Meet

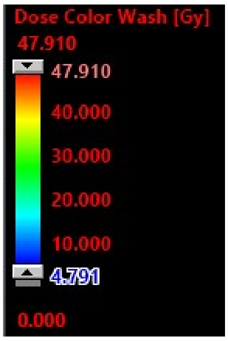

Above is reported the dose color wash of the figure 18d.

Patient_19

**N_isocenters = 2**	**Lesions = 47**
**N_fields = 6 (3+3)**	**Violated Constrains = target; PRV_SpinalCord ; PRV_CaudaEquina**

23a. 23b. 23c. 23d.

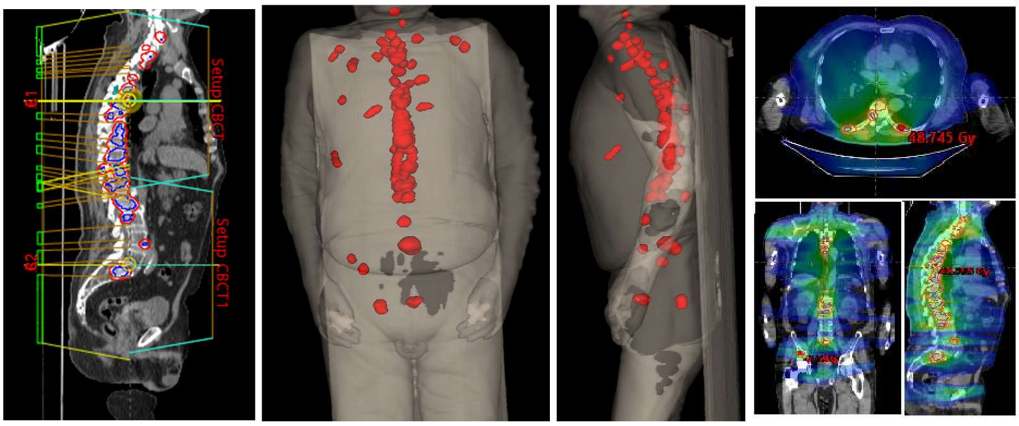

The figure above illustrates the number of isocenters (Fig.23a) used and the lesions spread in the body in the coronal (Fig 23b) and sagittal (Fig 23c) view by highlighting the PTV_all. Fig 23d shows the isodose levels in the three dimensions. For the isodose levels legend see below on the right.

The Table 26 below on the left reports the dose volume endpoints, indicating which the clinical goals were violated.

Dose Volume Endpoints			
**Structure**	**Clinical Goal**	**Value**	**Statutus**
**GTV_all**	V 110.0% > 95%	91.79 %	Violated
	123% < D 0.1 cm^3 < 126%	126.19 %	Violated
**PTV_all**	1.00 ≤ CI < 1.20	1.09	Meet
	V 100.0% > 95%	95.58 %	Meet
**Bladder**	D 15.0 cm^3	6.83 Gy	Meet
	D 0.1 cm^3	28.73 Gy	Meet
**Bowel**	D 0.1 cm^3	18.17 Gy	Meet
	D 20 cm^3	13.43 Gy	Meet
**BrachialPlexus_L**	D 3.0 cm^3	24.54 Gy	Meet
	D 0.1 cm^3	28.25 Gy	Meet
**BrachialPlexus_R**	D 3.0 cm^3	24.14 Gy	Meet
	D 0.1 cm^3	30.55 Gy	Meet
**BronchialTree**	D 5.0 cm^3	15.09 Gy	Meet
	D 0.1 cm^3	25.48 Gy	Meet
**Duodenum**	D 10 cm^3	16.49 Gy	Meet
	D 0.1 cm^3	20.21 Gy	Meet
**Esophagus**	D 0.1 cm^3	34.55 Gy	Meet
**FemurHead_L**	D 10 cm^3	13.46 Gy	Meet
**FemurHead_R**	D 10 cm^3	12.13 Gy	Meet
**GreatVessels**	D 0.1 cm^3	39.21 Gy	Meet
**Heart**	D 0.1 cm^3	25.66 Gy	Meet
	D 15.0 cm^3	19.81 Gy	Meet
**Kidneys – GTV_all**	D 200.0 cm^3	8.01 Gy	Meet
**Liver – GTV_all**	Dmean	10.20 Gy	Meet
	D 700 cm^3	9.03 Gy	Meet
**Lungs – GTV_all**	V 5.00 Gy	99.82 %	Violated
	V 20.00 Gy	6.38 %	Meet
	V13.50 Gy	19.01 %	Meet
	D 1500.0 cm^3	9.39 Gy	Meet
**PRV_CaudaEquina**	D 0.1 cm^3	37.04 Gy	Violated
**PRV_SpinalCord**	D 0.1 cm^3	38.61 Gy	Violated
**Rectum**	D 0.1 cm^3	17.88 Gy	Meet
**Stomach**	D 0.1 cm^3	18.36 Gy	Meet
	D 10 cm^3	14.40 Gy	Meet
**Trachea**	D 0.1 cm^3	30.45 Gy	Meet

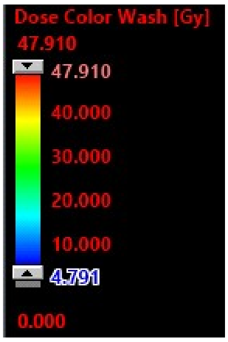

Above is reported the dose color wash of the figure 23d.

g. Monitor Units Reduction

Below, it is report a figure (Figure 24) that shows the plans MU value for each dose/fraction level, i.e. , their modification though the process of dose fraction reduction that was performed directly until the clinical goals were met. This reduction was performed without modifying the priority list in the optimizer. For each MU reduction level, the dose values of the dose-limiting OARs are reported. In addition, for each dose level, the table illustrates which OARs' constrains are violated and which are meet (i.e., ✗ : Violated - ✓ : Respected). The MU value of the level in which all clinical goals are met is highlighted in yellow. The illustration is figure is presented in the main manuscript.

Figure 24

**Patient**	**OARs**		**5x7Gy**		**5x6Gy**		**5x5Gy**		**5x4Gy**		**5x3Gy**		**5x2Gy**	
			MU: 9357.1		MU: 8020.4		MU: 6683.6		MU: 5346.8		MU: 4010.3		MU: 2673.5	
Patient_6	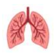	V5.0 Gy	97.51 %	✗	94.92 %	✗	92.45 %	✗	89.97 %	✗	85.18 %	✗	65.24 %	✓
		V20.0 Gy	54.36 %	✗	37.54 %	✗	20.71 %	✗	5.88 %	✓	0.00 %	✓	0.00 %	✓
		V13.5 Gy	80.35 %	✗	72.34 %	✗	59.37 %	✗	36.17 %	✓	12.23 %	✓	0.00 %	✓
		D1500 cc	24.47 Gy	✗	20.97 Gy	✗	17.48 Gy	✗	13.98 Gy	✗	10.49 Gy	✓	6.99 Gy	✓
			MU: 4494.3		MU: 3852.3		MU: 3210.2		MU: 2568.2		MU: 1926.1		MU: 854.7	

Patient_8		V5.0 Gy	95.15 %	✗	94.40 %	✗	93.52 %	✗	91.87 %	✗	85.66 %	✗	58.23 %	✓
		V20.0 Gy	44.48 %	✗	30.23 %	✗	16.79 %	✗	5.21 %	✓	0.00 %	✓	0.00 %	✓
		V13.5 Gy	78.89 %	✗	67.90 %	✗	50.38 %	✗	29.20 %	✓	10.12 %	✓	0.00 %	✓
		D1500 cc	18.33 Gy	✗	15.71 Gy	✗	13.09 Gy	✗	10.47 Gy	✓	7.85 Gy	✓	5.24 Gy	✓
		D0.1 cc	33.22 Gy	✗	28.47 Gy	✓	23.73 Gy	✓	18.98 Gy	✓	14.24 Gy	✓	5.24 Gy	✓
		D10.0 cc	18.33 Gy	✓	15.71 Gy	✓	13.09 Gy	✓	10.48 Gy	✓	7.86 Gy	✓	9.49 Gy	✓
			MU: 5437.9		MU: 4625.1		MU: 3883.4		MU: 3107.5					
Patient_9		V5.0 Gy	56.14 %	✓	54.79 %	✓	52.96 %	✓	50.26 %	✓				
		V20.0 Gy	30.45 %	✗	22.24 %	✗	13.04 %	✓	4.03 %	✓				
		V13.5 Gy	43.49 %	✗	38.89 %	✗	32.79 %	✓	21.55 %	✓				
		D1500 cc	18.88 Gy	✗	16.18 Gy	✗	13.48 Gy	✗	10.79 Gy	✓				
			MU: 13707.9		MU: 11749.9		MU: 9791.4		MU: 7833.1					
Patient_12		V5.0 Gy	92.96 %	✗	88.44 %	✗	81.64 %	✗	74.14 %	✓				
		V20.0 Gy	36.47 %	✗	23.99 %	✗	11.17 %	✓	2.94 %	✓				
		V13.5 Gy	59.69 %	✗	52.34 %	✗	40.86 %	✗	22.89 %	✓				
		D1500 cc	15.63 Gy	✗	13.39 %	✗	11.16 Gy	✓	8.93 Gy	✓				
		D0.1 cc	40.76 Gy	✗	34.93 Gy	✓	29.11 Gy	✓	23.29 Gy	✓				
		D15.0 cc	34.14 Gy	✗	29.26 Gy	✓	24.39 Gy	✓	19.51 Gy	✓				
		D5.00 cc	35.74 Gy	✓	30.63 Gy	✓	25.53 Gy	✓	18.70 Gy	✓				
		D0.1 cc	32.72 Gy	✗	28.08 Gy	✓	23.37 Gy	✓	1.06 Gy	✓				
			MU: 12714.6		MU: 10898.2									
Patient_14		Dmean Gy	17.62 Gy	✓	15.10 Gy	✓								
		D700.0 cc	17.23 Gy	✗	14.77 Gy	✓								
		D0.1 cc	33.36 Gy	✗	28.60 Gy	✓								
		D10.0 cc	16.08 Gy	✓	13.78 Gy	✓								
		D10.0 cc	30.35 Gy	✗	26.01 Gy	✓								
			MU 6348.1		MU: 5441.4		MU: 4534.3		MU: 3627.6		MU: 2720.7			
Patient_17		Dmean Gy	28.26 Gy	✗	24.22 Gy	✗	20.19 Gy	✗	16.15 Gy	✓	12.11 Gy	✓		
		D700.0 cc	32.28 Gy	✗	27.67 Gy	✗	23.06 Gy	✗	18.45 Gy	✗	13.83 Gy	✓		
		D0.1 cc	32.73 Gy	✗	28.06 Gy	✓	23.38 Gy	✓	18.71 Gy	✓	14.03 Gy	✓		
		D10.0 cc	22.02 Gy	✓	18.88 Gy	✓	15.73 Gy	✓	12.59 Gy	✓	9.44 Gy	✓		
			MU: 10390.7		MU: 8906.3		MU: 7422.1							
Patient_19		V5.0 Gy	99.82	✗	97.80 %	✗	79.44 %	✓						
		V20.0 Gy	6.38 %	✓	3.26 %	✓	0.97 %	✓						
		V13.5 Gy	19.01 %	✓	13.43 %	✓	7.78 %	✓						
		D1500 cc	9.39 %	✓	8.05 Gy	✓	6.71 Gy	✓						
		PRV_D0.1 cc	38.61 Gy	✗	33.09 Gy	✗	27.58 Gy	✓						
		PRV_D0.1 cc	37.04 Gy	✗	31.75 Gy	✗	26.46 Gy	✓						

LEGEND

Lungs-GTV_all	Stomach	Liver-GTV_all	Bronchial_Tree	FemurHead_R	Heart	PRV_SpinalCord	PRV_CaudaEquina

RPP dose-limiting OARs and their modification throughout direct dose reduction process. First column: patient identifier; second and third column: dose-limiting OAR and dose-limiting constraints identifier; other columns: constraints' values and status (i.e., ✗ : Clinical Goal Violated - ✓ : Clinical Goal Respected). Above each patient, the Monitor Unit of every dose level are reported and highlighted until all constraints are respected.

As performed in the table above, below we report the dose volume endpoints of the dose-limiting OARs for the two patients for whom it was possible to increase the prescribed dose, by modifying the objectives and priority list for each dose reduction level below 35 Gy (Figure 25). This was performed to evaluate whether it was possible to accept a higher RT schedule, by modifying the dose distribution for each dose level (i.e., a new optimization). For the other patients, it was not possible to achieve a higher prescription.

Figure 25

Patient	OARs	5x7Gy	5x6Gy	5x5Gy	5x4Gy

			MU: 5978		MU: 5613.1		MU: 4270.6		MU: 3416.5	
Patient_8		V5.0	92.49 %	✗	90.83 %	✗	86.33 %	✗	78.02 %	✓
		V20.0	24.93 %	✗	18.07 %	✗	12.22 %	✓	4.52 %	✓
		V13.5	50.27 %	✗	40.27 %	✗	27.26 %	✓	18.58 %	✓
		D1500	13.10 Gy	✗	12.06 Gy	✓	9.36 Gy	✓	7.49 Gy	✓
		D0.1	33.49 Gy	✗	28.83 Gy	✓	23.92 Gy	✓	19.14 Gy	✓
		D10.0	18.94 Gy	✗	16.90 Gy	✓	13.53 Gy	✓	10.82 Gy	✓
		D0.1	38.52 Gy	✗	31.43 Gy	✓	27.51 Gy	✓	22.01 Gy	✓
		D0.1	40.19 Gy	✗	32.19 Gy	✓	28.71 Gy	✓	22.96 Gy	✓
		D5.0	35.08 Gy	✗	29.63 Gy	✓	25.06 Gy	✓	20.05 Gy	✓
Patient_9			MU: 8212.3		MU: 7039.1					
		V5.0	52.87 %	✓	50.97 %	✓				
		V20.0	18.72 %	✗	14.44 %	✓				
		V13.5	33.67 %	✓	27.46 %	✓				
		D1500	13.78 Gy	✗	11.81 Gy	✓				

LEGEND

Lungs-GTV_all	Stomach	Liver-GTV_all	Bronchial_Tree	FemurHead_R	Heart

The figure shows the RPP Monitor Unit (MU) of the two patients for whom it was possible to achieve a higher prescribed dose, by modifying its distribution in the target for each level (i.e., a new optimization). For each dose/fraction level, i.e. and their modification though the process of dose fraction reduction that was performed until the clinical goals were met. This reduction was made without modifying the priority list in the optimizer. For the two patients, it is highlighted the OARs that resulted dose limiting (second column) and it is indicated for every which were the violated clinical goals (i.e., ✗ : Clinical Goal Violated - ✓ : Clinical Goal Respected).

h. Hematopoietic Bone Marrow

When large volumes of the central body are irradiated, the bone marrow, blood volume and lymph nodes may receive a significant dose to a large volume. This may seriously impact the patients hematopoietic cell compartment and immune system, counteracting the patient's possibility to receive systemic treatment and ultimately increased mortality. Thus, the dose received by the hematopoietic bone marrow may be a serious concern in these patients as it allows blood and immune cells turnover.

As the majority of hematopoietic bone marrow (HBM) in the adults is located in vertebral bodies, pelvis bones, and femoral heads, we assumed these structures as a reasonable patients' hematopoietic bone marrow approximation, as shown by the figure below.

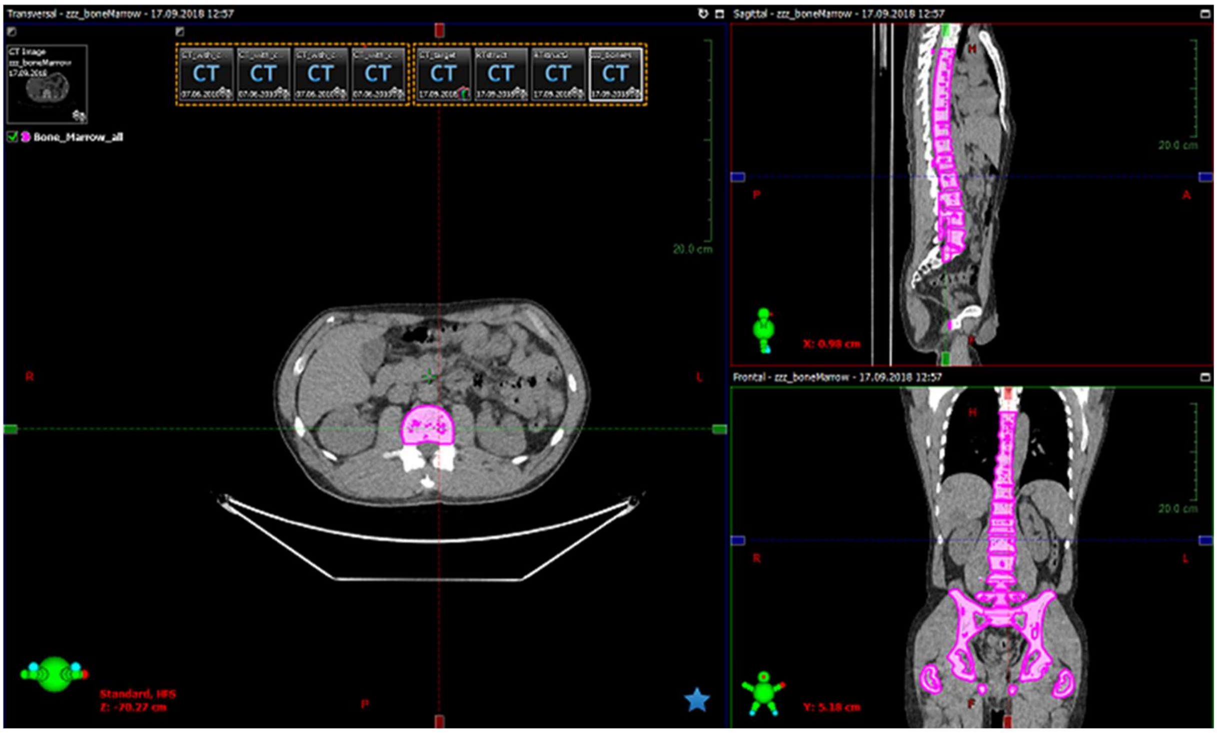

Considering the data on Total Body Irradiation (TBI), we decided to evaluate the Dmean and the V2Gy, as a single-dose nonmyeloablative TBI (2 Gy) seems not to be associated with significant acute side effects and it is well tolerated.

Below we report the data of each patient. The first series refer to Accepted Plans, while the second series refers to Rejected Plans.

ACCEPTED PLANS

Patient 1

V_tot = 1892 cc

V2 = 371 cc (19.6%)

Dmean = 3.4 Gy

Patient 2

V_tot = 1815 cc

V2 = 787 cc (43.3%)

Dmean = 2.9 Gy

Patient 3

V_tot = 1263 cc

V2 = 588 cc (46.5%)

Dmean = 3.9 Gy

Patient 4

V_tot = 1479 cc

V2 = 885 cc (59.8%)

Dmean = 5.2 Gy

Patient 5

V_tot = 1293 cc

V2 = 520 cc (40.2%)

Dmean = 3.9 Gy

Patient 7

V_tot = 1418 cc

V2 = 845 cc (59.6%)

Dmean = 7.4 Gy

Patient 10

V_tot = 1378 cc

V2 = 1052 cc (76.4%)

Dmean = 7.9 Gy

Patient 11

V_tot = 1939 cc

V2 = 1176 cc (60.7%)

Dmean = 4.6 Gy

Patient 13

V_tot = 1096 cc

V2 = 848 cc (77.3%)

Dmean = 10.8 Gy

Patient 15

V_tot = 1617 cc

V2 = 664 cc (41.1%)

Dmean = 3.5 Gy

Patient 16

V_tot = 1575 cc

V2 = 371 cc (23.6%)

Dmean = 2.9 Gy

Patient 18

V_tot = 1538 cc

V2 = 311 cc (20.2%)

Dmean = 1.6 Gy

Patient 20

V_tot = 1954 cc

V2 = 844 cc (43.2%)

Dmean = 4.5 Gy

Patient 21

V_tot = 1404 cc

V2 = 823 cc (58.6%)

Dmean = 4.5 Gy

Patient 22

V_tot = 1894 cc

V2 = 837 cc (44.2%)

Dmean = 4.2 Gy

Patient 23

V_tot = 998 cc

V2 = 409 cc (40.9%)

Dmean = 5.0 Gy

REJECTED PLANS

Patient 6

V_tot = 1748 cc

V2 = 1075 cc (61.5%)

Dmean = 7.6 Gy

Patient 8

V_tot = 1225 cc

V2 = 192 cc (15.7%)

Dmean = 2.6 Gy

Patient 9

V_tot = 1209 cc

V2 = 144 cc (11.9%)

Dmean = 1.7 Gy / 1.0 Gy

Patient 12

V_tot = 1932 cc

V2 = 446 cc (23.1%)

Dmean = 3.8 Gy

Patient 14

V_tot = 1667 cc

V2 = 1281 cc (76.9%)

Dmean = 8.4 Gy

Patient 17

V_tot = 1480 cc

V2 = 187 cc (12.7%)

Dmean = 2.1 Gy / 0.9 Gy

Patient 19

V_tot = 1671 cc

V2 = 1452 cc (86.9%)

Dmean = 15.8 Gy
