# Supplementary material for: An in-silico planning study of stereotactic body radiation therapy for polymetastatic patients with more than ten extra-cranial lesions
Source: Phys Imaging Radiat Oncol. 2024 Mar 3;30:100567. doi: 10.1016/j.phro.2024.100567 (PMC10950805; doi:10.1016/j.phro.2024.100567)
Supplement: Supplementary data 4 [file mmc4.pdf]

Supplementary_Material_D

The table below shows the clinical goals for both the target volumes and Organs At Risks used in Eclipse to optimize the 5-fraction SABR treatment plans.

TARGET	
**Structure**	**Clinical Goal**
**GTV_all**	V 110.0% > 95%
	D 0.1 cm^3 < 126% (Mandatory < 129%)
	D 0.1 cm^3 > 123%
**PTV_all**	CI ≥ 1.00
	CI < 1.20 (Mandatory < 1.40)
	V 100.0% > 95%
**OARs**	
**Structure**	**Clinical Goal in 5 fraction SBRT**
**Bladder**	D 15.0 cm^3 < 27.50 Gy
	D 0.1 cm^3 < 38.00 Gy
**Bowel**	D 0.1 cm^3 < 38.00 Gy
	D 20 cm^3 < 29.00 Gy
**BrachialPlexus_L**	D 3.0 cm^3 < 27.00 Gy
	D 0.1 cm^3 < 32.00Gy
**BrachialPlexus_R**	D 3.0 cm^3 < 27.00 Gy
	D 0.1 cm^3 < 32.00 Gy
**BronchialTree**	D 5.0 cm^3 < 32.00 Gy
	D 0.1 cm^3 < 38.00 Gy
**Duodenum**	D 10 cm^3 < 21.00 Gy
	D 0.1 cm^3 < 35.00 Gy
**Esophagus**	D 0.1 cm^3 < 35.00 Gy
**FemurHead_L**	D 10 cm^3 < 30.00 Gy
**FemurHead_R**	D 10 cm^3 < 30.00 Gy
**GreatVessel**	D 0.1 cm^3 < 53.00 Gy
**Heart**	D 0.1 cm^3 < 38.00 Gy
	D 15.0 cm^3 < 32.00 Gy
**Kidneys – GTV_all**	D 200.0 cm^3 < 17.50 Gy
**Liver – GTV_all**	Dmean < 18.00 Gy
	D 700 cm^3 < 15 Gy
**Lungs – GTV_all**	V 5.00 Gy < 80 %
	V 20 Gy < 15.0 %
	V 13.50 Gy < 37.00 %
	D 1500.0 cm^3 < 12.50 Gy
**PRV_CaudaEquina**	D 0.1 cm^3 < 31.50 Gy
**PRV_SpinaCord**	D 0.1 cm^3 < 28.00 Gy
**Rectum**	D 0.1 cm^3 < 38.00 Gy
**Stomach**	D 10 cm^3 < 25.00 Gy
	D 0.1 cm^3 < 32.00 Gy
**Trachea**	D cm^3 < 38.00 Gy
