# Supplementary material for: An in-silico planning study of stereotactic body radiation therapy for polymetastatic patients with more than ten extra-cranial lesions
Source: Phys Imaging Radiat Oncol. 2024 Mar 3;30:100567. doi: 10.1016/j.phro.2024.100567 (PMC10950805; doi:10.1016/j.phro.2024.100567)
Supplement: Supplementary data 5 [file mmc5.pdf]

Supplementary Material E

In Supplementary Material E, we discuss in more detail the methodology used for generating treatment plans using the cell-kill based approach, as well as the corresponding results for Patient_17. In addition, we also report a robustness analysis showing the impact of setup errors on the quality of the resulting plans.

Planning objectives and constraints

For maximum local consolidative radiotherapy of polymetastatic cancer patients, treatment planning is performed aiming to minimize the number of surviving tumor cells, given some OAR constraints. To this end, we considered the linear-quadratic (LQ) model for cell survival, as defined in Equation (1). For simplicity, the radiation sensitivity is assumed to be constant within each metastasis, i.e. $\alpha_i = \alpha$ and $\beta_i = \beta\ \forall i \in PTV_m$. In this way, it is possible to evaluate the effectiveness of a treatment plan using the equivalent uniform dose (EUD) concept previously proposed by Niemierko *et al*. The EUD is defined as the total radiation dose that, if delivered uniformly to all tumor voxels, leads to the same number of surviving tumor cells as a non-uniform dose distribution, i.e.

$$\sum_{i \in PTV\_all} \exp\left((-\alpha d_i - \beta d_i^2)N_f\right) = N_{PTV\_all} \exp\left((-\alpha EUD - \beta EUD^2)N_f\right)$$

where N_{PTV_all} is the number of voxels within the PTV. By solving for the EUD, this leads to

$$EUD = \frac{-\frac{\alpha}{\beta} + \sqrt{\left(\frac{\alpha}{\beta}\right)^2 - \frac{4}{\beta N_f} ln\left(\frac{1}{N_{PTV\_all}} \sum_{i \in PTV\_all} \exp\left((-\alpha d_i - \beta d_i^2)N_f\right)\right)}}{2}$$

The EUD has been implemented into our in-house optimization code as a planning objective. As the EUD is a strictly increasing function of tumor cell kill, maximizing the tumor EUD leads to a treatment plan that minimizes the number of surviving tumor cells. In this project, we assumed a typical α/β-ratio of 10 Gy for all PTV voxels, while α was set to 0.29 Gy^{-1}. The value for α was chosen such that only 50% of the tumor cells survive after receiving a dose of 2 Gy.

An additional planning objective was used to achieve an adequate dose conformity. This objective mimics the normal tissue objective used in the Eclipse TPS, which requires that the normal tissue dose falls off exponentially with increasing distance from the PTV edge. However, as no dose must be prescribed to the PTV with the cell-kill based approach, the EUD is used as reference dose for the normal tissue objective, which is defined as a quadratic penalty function:

$$f(\boldsymbol{d}) = \sum_{i \in NT} \frac{1}{N_{NT}} \left(d_i - d_{ref}(s_i)\right)^2$$

where NT specifies the set of voxels belonging to the normal tissue and

$$d_{ref}(s_i) = EUD\ (f_0 \exp(-ks_i) + f_\infty(1 - \exp(-ks_i))$$

is a voxel-specific maximum dose which depends on the distance s_i of voxel i from the PTV edge. In this study, we defined $f_0 = 0.95$, $f_\infty = 0.3$ and $k = 0.6$. As the EUD iteratively changes during the optimization, the reference penalty doses for this objective are also continuously updated.

To achieve the clinical goals for the OAR reported in Table 1, planning constraints have been defined for the optimization. Each constraint is handled using an augmented Lagrangian method [1]. In this way, no fine tuning of the objective values and priorities is needed to ensure that the clinical goals for the OARs are met.

Field choice and computational setup

For both patients Patient_6 and Patient_17, we generated a five-fraction IMRT plan using 35 coplanar 6 MV photon beams for each isocenter used in the corresponding Eclipse plans, equispaced at gantry angles of about 10°. This allows to approximate the VMAT arcs used in the plans generated using the Eclipse TPS. Calculation of the dose-influence matrix elements Dij is performed with the open-source radiotherapy planning platform CERR [2] using a quadrant infinite beam (QIB) algorithm [3]. The beamlet size was set to 5 x 5 mm for both patients, whereas the dose grid sizes were 1.95 x 1.95 x 3.00 mm^3 for Patient_6 and 2.27 x 2.27 x 3.27 mm^3 for Patient_17. To find a local minimum of the optimization problem, we used our own implementation of the L-BFGS quasi-Newton method [4].

Treatment plan obtained using the cell-kill based planning approach for patient M194

The dose-volume histograms and dose distribution achieved for patient M194 using the cell-kill based planning approach are illustrated in Figure C1. Similar minimum doses of approximately 12-13 Gy are delivered to all metastases in Patient_17, where the value of the minimum dose is limited by the dose constraints on the healthy lung. However, inhomogeneous doses are delivered within each lesion, with the mean and maximum doses varying in between the individual metastases up to 2.0 Gy and 8.0 Gy, respectively.

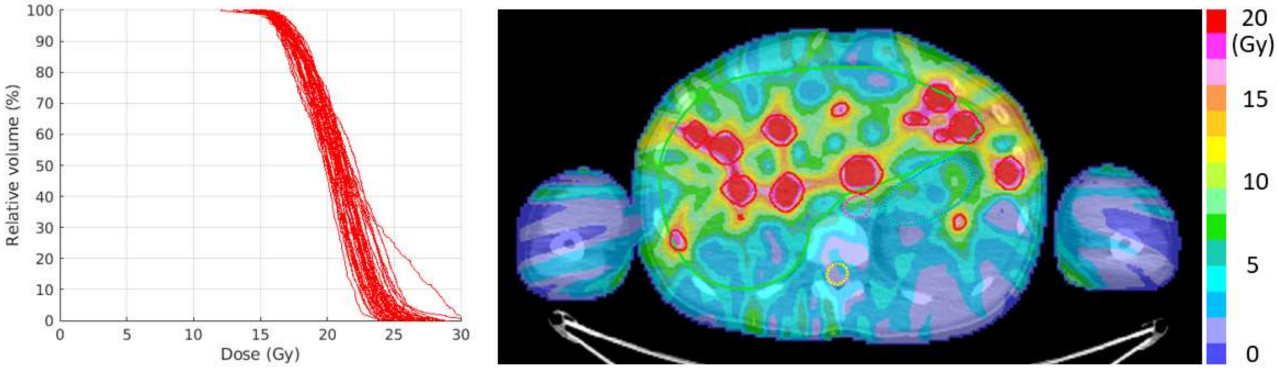

Figure C1: (a) Dose-volume histograms for the PTV of each metastasis in Patient_17. (b) Dose distribution obtained using the cell-kill based planning approach for Patient_17. The contours for the PTV (red), liver (green), stomach (light blue), great vessel (violet) and spinal cord (yellow) are also illustrated.

Sensitivity of treatment plans obtained using the cell-kill based approach to random setup errors

Figure C2 shows the dose-volume histograms for the GTV_all evaluated for both the nominal scenario (where no setup error is assumed) and for multiple error scenarios, which assume that rigid shifts in the GTV_all position of approximately ±6 mm apply in left–right, superior–inferior, and anterior–posterior directions between the different fractions. As the cell-kill based planning approach suggests to deliver a higher dose to the center of the metastases (i.e. at the position of the GTV_all in the nominal scenario), setup errors may result in a slight underdosage of the GTV_all dose compared to the situation where no setup error is assumed. Nevertheless, such setup uncertainties only have a little impact

on the tumor EUD. The EUD to the GTV_all in the nominal scenario is 3.3 Gy for Patient_6 and 4.4 Gy for Patient_17, respectively. Considering all evaluated error scenarios, the EUD to the GTV_all reads 3.2±0.1 Gy (with a EUD of 2.7 Gy for the worst case scenario) for Patient_6 and 4.2±0.1 Gy (with a EUD of 3.8 Gy for the worst case scenario) for Patient_17.

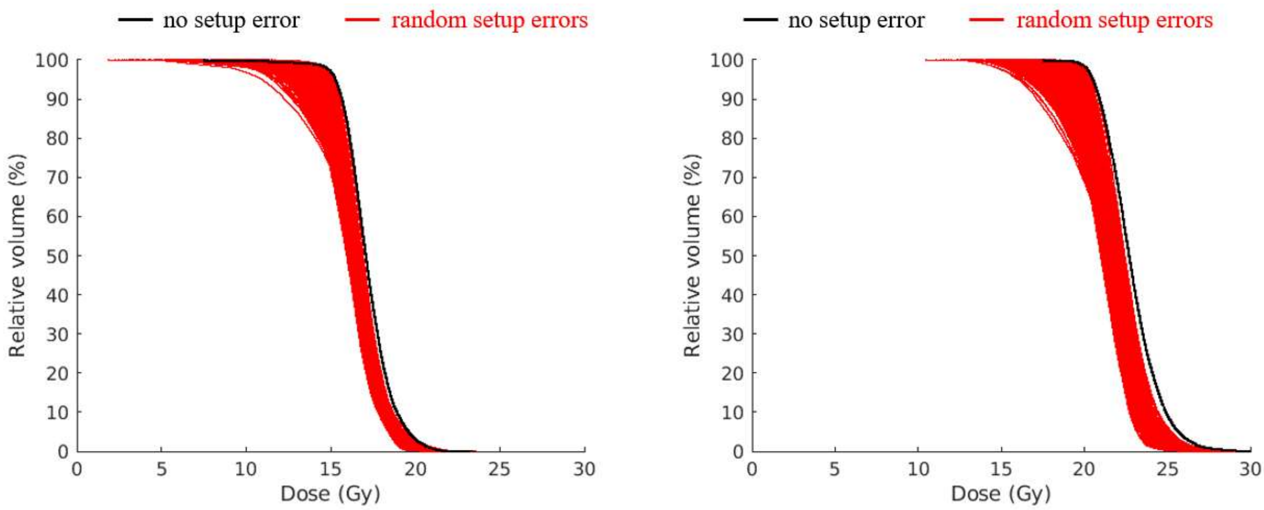

(a) Patient_6 (b) Patient_17

Figure C2: Dose–volume histograms for the GTV_all in (a) Patient_6 and (b) Patient_17 evaluated assuming a scenario with no setup errors (black line) and multiple error scenarios corresponding to all combinations of random setup errors of ±6 mm in left–right, superior–inferior, and anterior–posterior directions (red lines).

REFERENCES

[1] Bertsekas DP. Nonlinear Programming. Athena Scientific; 1999.

[2] Deasy JO, Blanco AI, Clark VH. CERR: a computational environment for radiotherapy research. Med Phys. 2003 May;30(5):979-85. doi: 10.1118/1.1568978. PMID: 12773007.

[3] Kalinin EDJ. A method for fast 3-D IMRT dose calculations the quadrant infinite beam (QIB) algorithm. Paper presented at: 45th Annual Meeting of the American Association of Physicist in Medicine; 2003.

[4] Wright SJ, Nocedal J. Numerical Optimization. Vol 2. Springer; 1999.
