# Supplementary material for: An in-silico planning study of stereotactic body radiation therapy for polymetastatic patients with more than ten extra-cranial lesions
Source: Phys Imaging Radiat Oncol. 2024 Mar 3;30:100567. doi: 10.1016/j.phro.2024.100567 (PMC10950805; doi:10.1016/j.phro.2024.100567)
Supplement: Supplementary data 6 [file mmc6.pdf]

Appendix_F

AP vs RP

Below we report the comparison between the accepted plans (AP) and the rejected plans (RP), considering the patients' characteristics, the metastases number and their disposition across the body as well as the infiltrated structures. For each data-point is reported if the difference between AP and RP was statistically significant (p-value). We performed both parametric and non-parametric tests. Please, see next lines for further information about how the statistical analysis was performed for each variable.

	AP	RP	p-value
**Patients Characteristics**			
Patients Number	16	7	-
Age	64 years (38 – 83 years)	70 years (33-78 years)	p > 0.05
Sex	12 males – 5 females	5 males – 2 females	p > 0.05
Median Lesions Number	15 (range 11-28)	26 (range 11-51)	p < 0.05
**Lesions Number and Anatomical Disposition**			
Total Number	263 (100 %)	214 (100%)	-
H&N	8 (3.04 %)	12 (5.61%)	p > 0.05
Thorax	107 (40.68%)	100 (46.73%)	p > 0.05
Abdomen	84 (31.94%)	84 (39.25%)	p > 0.05
Pelvis	32 (12.17%)	12 (5.61%)	p > 0.05
Extremity	32 (12.17%)	6 (2.80%)	p > 0.05
**Involved Anatomical Sites**			
Adrenal gland	12 (4.56 %)	1 (0.47 %)	p > 0.05
Bone	29 (11.03 %)	64 (29.91 %)	p > 0.05
Heart	0 (0.00 %)	1 (0.47 %)	p > 0.05
Intercostal	1 (0.38%)	0 (0.00 %)	p > 0.05
Intraperitoneal	12 (4.56%)	1 (0.47 %)	p > 0.05
Lymph nodes	68 (25.86 %)	27 (12.62 %)	p > 0.05
Liver	32 (12.71%)	59 (27.57 %)	p > 0.05
Lung	51 (19.39%)	53 (24.77 %)	p > 0.05
Muscle	8 (3.04%)	0 (0.00 %)	p > 0.05
Retroperitoneal	7 (2.66 %)	0 (0.00 %)	p > 0.05
Spleen	4 (1.52%)	7 (3.27%)	p > 0.05
Subcutaneous	22 (8.37%)	1 (0.47 %)	p > 0.05
Paravertebral	17 (6.46 %)	0 (0.00 %)	p > 0.05
**Plans Characteristics**			
Median Treated Volume	171.30 cc (118.2 – 435.1 cc)	497.43 cc (235.84 – 756.7 cc)	p < 0.05
Median MU	6937.95 (3390.4 – 9570.4)	9357.1 (4494.3 – 13807.9)	p > 0.05
Median Arcs Number	5 (2-8)	6 (3-8)	p > 0.05
Median Isocenters Number	2 (1-3)	2 (1-3)	p > 0.05
Median Infiltrated Lungs' Volume	0.03 % (0.00 – 1.07 %)	0.12 % (0.00 – 2.81 %)	p > 0.05
Median Treated Lungs' Volume*	0.44 % (0.00 – 1.40 %)	2.29 % (0.00 - 6.66 %)	p > 0.05
Median Infiltrated Liver's Volume	0.28 % (0.00 – 5.44 %)	0.01 % (0.00 – 5.01 %)	p > 0.05
Median Treated Liver's Volume*	1.30 % (0.00 – 10.02 %)	0.27 % (0.00 – 20.74 %)	p > 0.05

The p-values were calculated by performing the Mann-Whitney U Test with a significance level of 0.05 under the two-tailed hypothesis. Additionally, we calculated also the z-score for each variable and its p-value, however, it is noteworthy that the approximation to the form of the normal distribution becomes less robust for sample sizes smaller than 10, so caution is appropriate here in the interpretation and use of the Z-value calculation. For categorical variable, e.g., SEX, we performed the Chi-Square Test to obtain the p-value.

- Sample 1 – AP (16 patients)
- Sample 2 – RP (7 patients)

Patients' Characteristics

A. AGE

The U-value is 53. The critical value of U at $p < 0.05$ is 26. Therefore, the result is not significant at $p < 0.05$.

The z-score is -0.16704. The p-value is 0.86502. The result is not significant at $p < 0.05$.

B. SEX

The chi-square statistic is 0.0322. The p-value is 0.857561. The result is not significant at $p < 0.05$.

C. LESIONS NUMBER

The U-value is 21. The critical value of U at $p < 0.05$ is 26. Therefore, the result is significant at $p < 0.05$.

The z-score is -2.30513. The p-value is 0.02088. The result is significant at $p < 0.05$.

Lesions Number and Anatomical Disposition

A. H&N

The U-value is 52. The critical value of U at $p < 0.05$ is 26. Therefore, the result is not significant at $p < 0.05$.

The z-score is -0.23385. The p-value is 0.8181. The result is not significant at $p < 0.05$.

B. THORAX

The U-value is 31. The critical value of U at $p < 0.05$ is 26. Therefore, the result is not significant at $p < 0.05$.

The z-score is -1.63698. The p-value is 0.101. The result is not significant at $p < 0.05$.

C. ABDOMEN

The U-value is 51. The critical value of U at $p < 0.05$ is 26. Therefore, the result is not significant at $p < 0.05$.

The z-score is -0.30067. The p-value is 0.76418. The result is not significant at $p < 0.05$.

D. PELVIS

The U-value is 48.5. The critical value of U at $p < 0.05$ is 26. Therefore, the result is not significant at $p < 0.05$.

The z-score is 0.46771. The p-value is 0.63836. The result is not significant at $p < 0.05$.

E. EXTREMITY

The U-value is 51. The critical value of U at $p < 0.05$ is 26. Therefore, the result is not significant at $p < 0.05$.

The z-score is -0.30067. The p-value is 0.76418. The result is not significant at $p < 0.05$.

Involved Anatomical Sites

A. ADRENAL GLAND

The U-value is 34. The critical value of U at $p < 0.05$ is 26. Therefore, the result is not significant at $p < 0.05$.

The z-score is 1.43653. The p-value is 0.14986. The result is not significant at $p < 0.05$

B. BONE

The U-value is 49.5. The critical value of U at $p < 0.05$ is 26. Therefore, the result is not significant at $p < 0.05$.

The z-score is -0.40089. The p-value is 0.68916. The result is not significant at $p < 0.05$.

C. HEART

The U-value is 48. The critical value of U at $p < 0.05$ is 26. Therefore, the result is not significant at $p < .05$.

The z-score is -0.50111. The p-value is 0.61708. The result is not significant at $p < .05$.

D. INTERCOSTAL

The U-value is 52.5. The critical value of U at $p < 0.05$ is 26. Therefore, the result is not significant at $p < 0.05$.

The z-score is 0.20045. The p-value is 0.84148. The result is not significant at $p < 0.05$.

E. INTRAPERITONEAL

The U-value is 42. The critical value of U at $p < 0.05$ is 26. Therefore, the result is not significant at $p < 0.05$.

The z-score is 0.90201. The p-value is 0.36812. The result is not significant at $p < 0.05$.

F. LYMPH NODES

The U-value is 52.5. The critical value of U at $p < 0.05$ is 26. Therefore, the result is not significant at $p < 0.05$.

The z-score is 0.20045. The p-value is 0.84148. The result is not significant at $p < 0.05$

G. LIVER

The U-value is 56. The critical value of U at $p < 0.05$ is 26. Therefore, the result is not significant at $p < 0.05$.

The z-score is 0.03341. The p-value is 0.97606. The result is not significant at $p < 0.05$.

H. LUNG

The U-value is 46.5. The critical value of U at $p < 0.05$ is 26. Therefore, the result is not significant at $p < 0.05$.

The z-score is -0.60134. The p-value is 0.5485. The result is not significant at $p < 0.05$.

I. MUSCLE

The U-value is 38.5. The critical value of U at $p < 0.05$ is 26. Therefore, the result is not significant at $p < 0.05$.

The z-score is 1.13586. The p-value is .25428. The result is not significant at $p < 0.05$.

L. RETROPERITONEAL

The U-value is 42. The critical value of U at $p < 0.05$ is 26. Therefore, the result is not significant at $p < 0.05$.

The z-score is 0.90201. The p-value is 0.36812. The result is not significant at $p < 0.05$.

M. SPLEEN

The U-value is 54. The critical value of U at $p < 0.05$ is 26. Therefore, the result is not significant at $p < 0.05$.

The z-score is -0.10022. The p-value is 0.92034. The result is not significant at $p < 0.05$.

N. SUBCUTANEOUS

The U-value is 49. The critical value of U at $p < .05$ is 26. Therefore, the result is not significant at $p < 0.05$.

The z-score is 0.4343. The p-value is 0.6672. The result is not significant at $p < 0.05$.

O. PARAVERTEBRAL

The U-value is 45.5. The critical value of U at $p < 0.05$ is 26. Therefore, the result is not significant at $p < 0.05$.

The z-score is 0.66815. The p-value is 0.50286. The result is not significant at $p < 0.05$.

Plans Characteristics

A. GTV_all volume

The U-value is 23. The critical value of U at $p < 0.05$ is 26. Therefore, the result is significant at $p < 0.05$.

The z-score is -2.1715. The p-value is 0.03. The result is significant at $p < 0.05$.

B. MEDIAN TREATED VOLUME

The U-value is 13. The critical value of U at $p < 0.05$ is 26. Therefore, the result is significant at $p < 0.05$.

The z-score is -2.83965. The p-value is 0.00452. The result is significant at $p < 0.05$.

C. MEDIAN MONITOR UNITS

The U-value is 37. The critical value of U at $p < 0.05$ is 26. Therefore, the result is not significant at $p < 0.05$.

The z-score is -1.23608. The p-value is 0.21498. The result is not significant at $p < 0.05$.

D. MEDIAN ARCS NUMBER

The U-value is 39.5. The critical value of U at $p < 0.05$ is 26. Therefore, the result is not significant at $p < 0.05$.

The z-score is -1.06904. The p-value is 0.28462. The result is not significant at $p < 0.05$.

E. MEDIAN ISOCENTERS NUMBER

The U-value is 49. The critical value of U at $p < 0.05$ is 26. Therefore, the result is not significant at $p < 0.05$.

The z-score is -0.4343. The p-value is 0.6672. The result is not significant at $p < 0.05$.

F. MEDIAN INFILTRATED LUNGS' VOLUME

The U-value is 42. The critical value of U at $p < 0.05$ is 26. Therefore, the result is not significant at $p < 0.05$.

The z-score is -0.90201. The p-value is 0.36812. The result is not significant at $p < 0.05$.

G. MEDIAN TRATED LUNGS' VOLUME

The U-value is 36. The critical value of U at $p < 0.05$ is 26. Therefore, the result is not significant at $p < 0.05$.

The z-score is -1.3029. The p-value is 0.1936. The result is not significant at $p < 0.05$.

H. MEDIAN INFILTRATED LIVER'S VOLUME

The U-value is 49.5. The critical value of U at $p < 0.05$ is 26. Therefore, the result is not significant at $p < 0.05$.

The z-score is 0.40089. The p-value is 0.68916. The result is not significant at $p < 0.05$.

I. MEDIAN TREATED LIVERS'S VOLUME

The U-value is 53. The critical value of U at $p < 0.05$ is 26. Therefore, the result is not significant at $p < 0.05$.

The z-score is 0.16704. The p-value is .86502. The result is not significant at $p < 0.05$.
