# Supplementary material for: An in-silico planning study of stereotactic body radiation therapy for polymetastatic patients with more than ten extra-cranial lesions
Source: Phys Imaging Radiat Oncol. 2024 Mar 3;30:100567. doi: 10.1016/j.phro.2024.100567 (PMC10950805; doi:10.1016/j.phro.2024.100567)
Supplement: Supplementary data 7 [file mmc7.pdf]

Appendix_G

Gamma agreement analysis for patient 1

The plan was irradiated on the Delta4 phantom (Scandidos, Sweden). A global gamma agreement analysis was performed on the two detector planes using the Software provided with the phantom. A threshold of 20% was used, doses below the threshold were not evaluated.

Longitudinal profiles in the two detector planes of the Delta4 phantom.

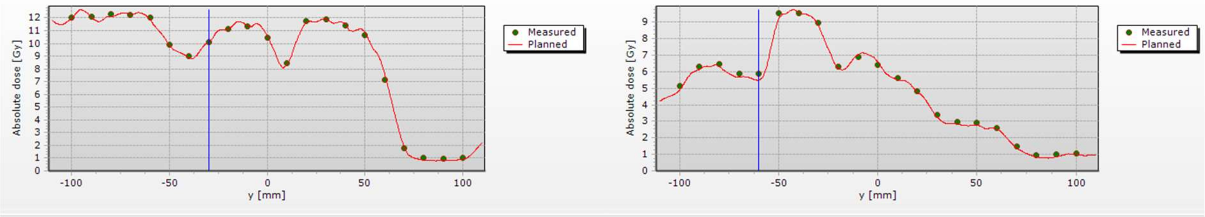

Dose deviation, distance to agreement and gamma agreement index (3%, 2mm) for the total plan:

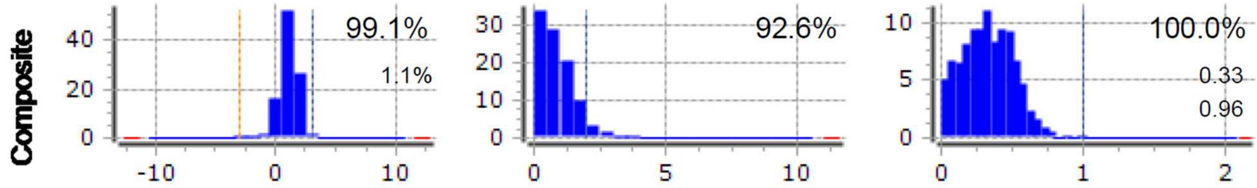

Global gamma agreement index for different DTA and Dose deviations criteria:

Gamma Index Evaluations

Pass Rates [%] — Reference: Planned dose — Composite

DTA	0.5 %	1.0 %	1.5 %	2.0 %	2.5 %	3.0 %	3.5 %	4.0 %	4.5 %	5.0 %
5.0 mm	95.4	98.0	99.6	100.0	100.0	100.0	100.0	100.0	100.0	100.0
4.5 mm	94.4	97.4	99.4	100.0	100.0	100.0	100.0	100.0	100.0	100.0
4.0 mm	91.7	96.0	99.0	99.9	100.0	100.0	100.0	100.0	100.0	100.0
3.5 mm	87.3	94.4	98.5	99.7	100.0	100.0	100.0	100.0	100.0	100.0
3.0 mm	82.4	91.0	97.9	99.3	99.9	100.0	100.0	100.0	100.0	100.0
2.5 mm	75.8	86.0	96.5	99.0	99.9	100.0	100.0	100.0	100.0	100.0
2.0 mm	68.1	79.9	93.6	98.5	99.9	100.0	100.0	100.0	100.0	100.0
1.5 mm	57.3	71.7	89.9	98.0	99.8	100.0	100.0	100.0	100.0	100.0
1.0 mm	40.7	61.1	83.1	95.8	99.3	99.9	100.0	100.0	100.0	100.0
0.5 mm	26.6	48.7	74.3	92.5	98.4	99.6	99.8	99.9	100.0	100.0

Dose Deviation
